# Systemic elevation of gut derived *p*-cresol during one year in space: implications for brain, heart, bone, liver and microbiome in astronauts

**DOI:** 10.3389/fphys.2026.1878680

**Published:** 2026-09-28

**Authors:** Michael A. Schmidt, Caleb M. Schmidt, Julian C. Schmidt, Cem Meydan, Evan E. Afshin, Eliah G. Overbey, JangKeun Kim, Ruth Subhash Singh, Afshin Beheshti, Christopher E. Mason

**Affiliations:** 1 Sovaris Aerospace, Boulder, CO, United States; 2 Department of Medicine, Aerospace Medicine, University of Central Florida College of Medicine, Orlando, FL, United States; 3 Center for Aerospace and Extreme Environments Medicine, UCF College of Medicine, Orlando, FL, United States; 4 Department of Systems Engineering, Colorado State University, Fort Collins, CO, United States; 5 Division of Hematology and Medical Oncology, Sanford I. Weill Department of Medicine, Weill Cornell Medicine, New York, NY, United States; 6 Department of Physiology and Biophysics, Weill Cornell Medicine, New York, NY, United States; 7 The HRH Prince Alwaleed Bin Talal Bin Abdulaziz Alsaud Institute for Computational Biomedicine, Weill Cornell Medicine, New York, NY, United States; 8 Center for STEM, University of Austin (UATX), Austin, TX, United States; 9 Department of Biomedical Engineering, Rensselaer Polytechnic Institute, Troy, NY, United States; 10 Stanley Center for Psychiatric Research, Broad Institute of MIT and Harvard, Cambridge, MA, United States; 11 Center for Space Biomedicine, Trivedi Institute for Space and Global Biomedicine, University of Pittsburgh School of Medicine, Pittsburgh, PA, United States; 12 McGowan Institute for Regenerative Medicine, Department of Surgery, University of Pittsburgh, Pittsburgh, PA, United States; 13 Computational and Systems Biology, University of Pittsburgh School of Medicine, Pittsburgh, PA, United States; 14 Trivedi Institute for Space and Global Biomedicine, University of Pittsburgh School of Medicine, Pittsburgh, PA, United States; 15 The WorldQuant Initiative for Quantitative Prediction, Weill Cornell Medicine, New York, NY, United States; 16 The Feil Family Brain and Mind Research Institute, Weill Cornell Medicine, New York, NY, United States

**Keywords:** microbiome, multiomics, NASA twins study, *p*-cresol, precision medicine, spaceflight, metabolome, prebiotics

## Abstract

**Introduction:**

We observed the elevation of *p*-cresol in the untargeted metabolome of the multiomics portion of the NASA Twins Study of One Year in Space. *p*-Cresol is a uremic and neurotoxin that is produced via the metabolism of dietary tyrosine by gut bacteria. Intestinally produced *p*-cresol is absorbed into systemic circulation where it exerts its adverse metabolic effects and drives phenotypic changes relevant to astronauts, including the brain, heart, bone, liver, kidney, and colon.

**Methods:**

A pair of monozygotic twins were studied for 25 months, during which one subject spent 340 days aboard the International Space Station (ISS), while his identical twin remained on Earth. Molecular analyses included genome, epigenome, transcriptome, proteome, metabolome, immunome, and microbiome. Our metabolite feature extraction was focused on plasma *p*-cresol (*p*-cresyl sulfate [pCS] and *p*-cresol glucuronide [pCG]), along with associated biological dynamics that govern *p*-cresol production and metabolism.

**Results:**

Significance was observed for pCG when comparing Post-flight vs. Inflight (adj p = 0.016). pCS values were significantly altered Post-flight vs. Inflight (adj p = 0.002) and Post-flight vs. Late-flight (adj p = 0.037). pCS values for Early-flight vs. Pre-flight approached significance (adj p = 0.08). In alignment with elevated plasma *p*-cresol values at certain stages of spaceflight, we found an increase in count-normalized abundance (raw reads) for the major *p*-cresol producer *Clostridioides difficile* in TW (Flight) vs. HR (Ground) for Post-flight, Inflight, and Overall (adj p = 0.014, adj p = 0.0006, and adj p = 2.02x10¯^5^, respectively). Concomitantly, we observed a significant increase in count-normalized abundance for another of the major *p*-cresol producers *Olsenella uli* in TW vs. HR for Post-flight, Inflight, and Overall (adj p = 0.0045, adj p = 0.00043, adj p = 0.0016, respectively), including Interaction for Inflight vs. Pre-flight (adj p = 0.028).

**Discussion:**

We observed an elevation of *p*-cresol during one year in space, which may have significant implications for astronaut health and performance. Molecular findings and *p*-cresol countermeasure candidates are discussed. We also explore the process from untargeted molecular feature extraction to description of biological meaning, to new hypothesis generation, to plausible countermeasure development for human spaceflight.

## 1 Background

The human intestinal microbiota is well known for its complex effects on host metabolic phenotypes. However, the dynamics of human-microbial co-metabolism in long-duration human spaceflight is not well understood. The NASA Twins Study represents the longest continuous study of a human in space that incorporates longitudinal measures derived from multi-scale omics and utilizing a ground-based control. The study incorporated over 300 samples collected across 19 timepoints before, during, and after 340 days in space. Data were generated using multiple modalities to examine human and microbial biology, including isolation of saliva, skin, plasma, stool, urine, peripheral blood mononuclear cells (PBMCs), and immune cells. *p*-Cresol (4-methylphenol; para-methylphenol) and its two primary metabolites (pCS and pCG; **Figure 1**). These dynamics are of potential importance to human spaceflight because of their broad mechanistic dynamics and widespread effects on the human clinical phenotype, as established by an extensive body of research on Earth.

**Figure 1:**
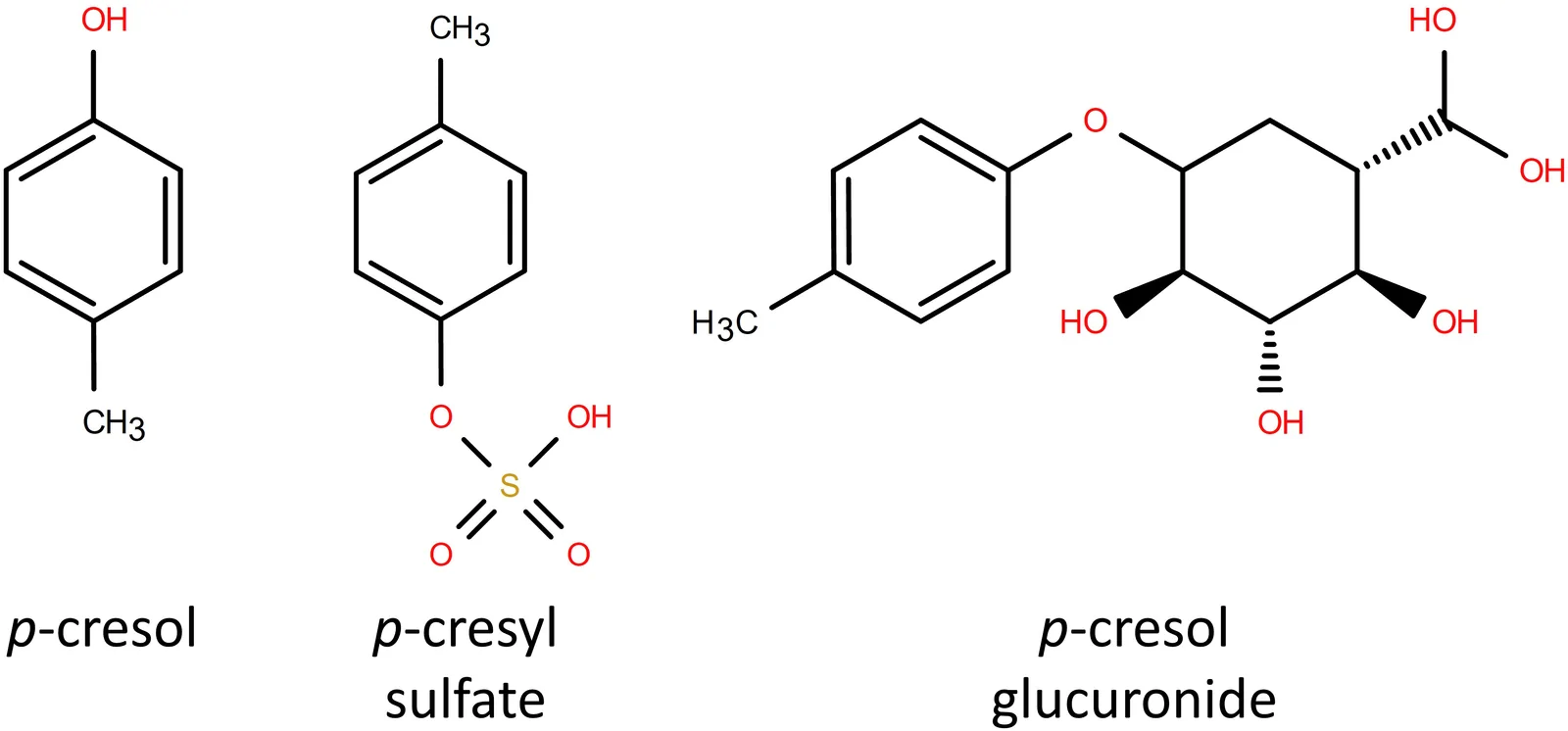
*p*-Cresol and its primary metabolites.

### 1.1 Biosynthesis of *p*-cresol

*p*-Cresol is produced in the gut via the metabolism of dietary tyrosine (**Figure 2**) by a range of bacterial families, such as *Bacteroidaceae*, Bifidobacteriaceae, Clostridiaceae, Enterobacteriaceae, Enterococcaceae, Eubacteriaceae, Fusobacteriaceae, Lachnospiraceae, Lactobacillaceae, Porphyromonadaceae, Staphylococcaceae, Ruminococcaceae, and *Veillonellaceae (*Gryp et al., 2020*)*. Previously, roughly 55 enteric species have been identified as *p*-cresol producers with the highest producers belonging to the specific family Coriobacteriaceae, or to Clostridium clusters XI and XIVa. Only *Blautia hydrogenotrophica* is known to produce *p*-cresol at comparable millimolar levels to that of clostridia (Saito et al., 2018).

**Figure 2:**
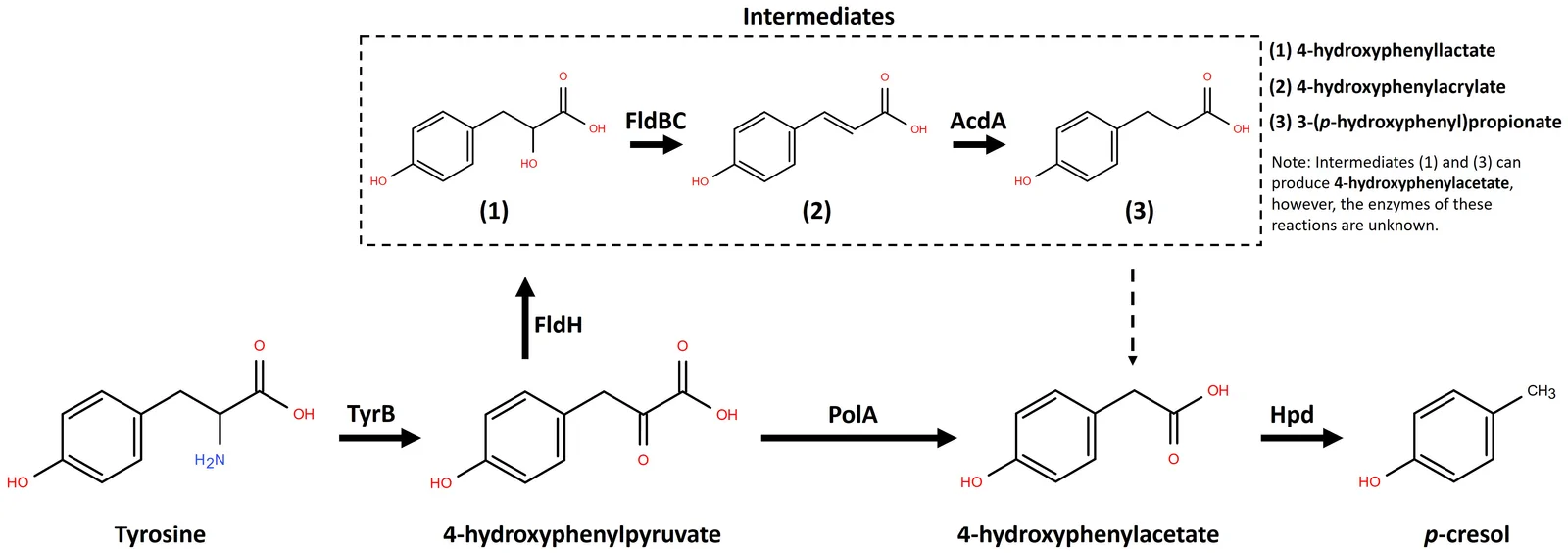
Synthesis of *p*-cresol from L-tyrosine.

*p*-Cresol in the colon is also known to suppress organisms that compete with clostridial species (major *p*-cresol producers), which contributes to clostridial survival and pathogenesis. In general, Gram-negative species are relatively sensitive to *p*-cresol, whereas Gram-positive species appear to be more tolerant. Disruption of the cell envelope is one mechanism by which *p*-cresol inhibits Gram-negative bacteria (Passmore et al., 2018). Among the organisms most sensitive to *p*-cresol are *Escherichia coli, Klebsiella oxytoca, and Bacteroides thetaiotaomicron*. Indeed, incubating ex vivo healthy human fecal samples with *p*-cresol, has shown a significant reduction in the number of viable total anaerobes, facultative anaerobes, and lactose fermenting enterobacteriacea.

Of particular importance is the capacity for *p*-cresol to inhibit the growth of butyrate producing organisms and reduce their attendant production of butyrate. These taxa, include but are not limited to *Eubacterium rectale*, *Faecalibacterium prausnitzii*, and *Roseburia* spp., which specialize in fermenting dietary fibers and resistant starches into butyrate (Singh et al., 2023). Butyrate is critical to gastrointestinal function due to its role as the preferred energy substrate for colonocytes (yielding around 70% of the cell’s energy) and its maintenance of gut barrier integrity. Butyrate also acts as a signaling molecule and regulates the immune response via histone deacetylase (HDAC) inhibition and G-protein-coupled receptor activation (Hodgkinson et al., 2023). These butyrate HDAC effects also extend to the central nervous system.

A better understanding of how *p*-cresol producing and butyrate producing microbes change in space will be central to explaining the *p*-cresol signal examined in our human study of one year in space. This begins with a basic understanding of known *p*-cresol producing and butyrate producing microbes on Earth, which is presented in **Table 1**.

**Table 1:** Summary of known *p*-cresol and butyrate producing microbes in the human gut.

*p*-Cresol producing microbes		
High *p*-cresol producers		
*Blautia hydrogenotrophica*	*Clostridioides difficile*	*Olsenella uli*
*Romboutsia lituseburensis*		
Low *p*-cresol producers		
*Bifidobacterium longum* *subsp. Infantis (B. infantis)*	*Anaerostipes caccae*	*Anaerostipes hadrus*
*Bacteroides caccae*	*Bacteroides dorei*	*Bacteroides ovatus*
*Bacteroides plebeius*	*Bacteroides uniformis*	*Bacteroides vulgatus*
*Clostridium bifermentans*	*Clostridium celerecrescens*	*Clostridium clostridioforme*
*Clostridium cochlearium*	*Clostridium ghonii*	*Clostridium glycolicum*
*Clostridium indolis*	*Clostridium innocuum*	*Clostridium leptum*
*Clostridium limosum*	*Clostridium nexile*	*Clostridium paraputrificum*
*Clostridium saccharolyticum*	*Clostridium scindens*	*Clostridium sordellii*
*Clostridium sphenoides*	*Clostridium symbiosum*	*Fusobacterium necrogenes*
*Fusobacterium varium*	*Ruminococcus gnavus*	*Ruminococcus torques*
*Veillonella parvula*		
Butyrate producing microbes		
High butyrate producers		
*Anaerobutyricum hallii*	*Anaerobutyricum soehngenii*	*Anaerostipes butyraticus*
*Anaerostipes caccae*	*Anaerostipes hadrus*	*Anaerotruncus colihominis*
*Butyrivibrio crossotus*	*Butyrivibrio fibrisolvens*	*Clostridium butyricum*
*Coprococcus catus*	*Coprococcus comes*	*Coprococcus eutactus*
*Eubacterium hallii*	*Eubacterium rectale*	*Faecalibacterium prausnitzii*
*Roseburia faecis*	*Roseburia hominis*	*Roseburia intestinalis*
*Roseburia inulinivorans*	*Ruminococcus bromii*	*Subdoligranulum variabile*
Low butyrate producers		
*Alistipes putredinis*	*Alistipes shahii*	*Anaerostipes rhamnosivorans*
*Blautia hydrogenotrophica*	*Blautia producta*	*Blautia wexlerae*
*Butyricicoccus pullicaecorum*	*Clostridium acetobutylicum*	*Clostridium beijerinckii*
*Clostridium tyrobutyricum*	*Dorea formicigenerans*	*Dorea longicatena*
*Eubacterium eligens*	*Eubacterium ramulus*	*Eubacterium ventriosum*
*Flavonifractor plautii*	*Fusobacterium nucleatum*	*Intestinimonas butyriciproducens*
*Lachnospira eligens*	*Lachnospira multipara*	*Megasphaera elsdenii*
*Megasphaera massiliensis*	*Oscillibacter valericigenes*	*Papillibacter cinnamivorans*
*Pseudobutyrivibrio ruminis*	*Ruminococcus albus*	*Ruminococcus callidus*
*Ruminococcus champanellensis*	*Ruminococcus flavefaciens*	*Shuttleworthia satelles*

Adapted from (Saito et al., 2018); (Gryp et al., 2020); and from (Singh et al., 2023).

### 1.2 Absorption and metabolism of *p*-cresol

Once produced from colonic microbial metabolism, *p*-cresol is absorbed by colonocytes, where it undergoes first pass metabolism. First pass metabolism consists of formation of pCS and pCG, some of which occurs prior to their release into the portal vein (Aronov et al., 2011). *p*-Cresol that has not been metabolized by colonocytes can undergo conjugation to form pCS and pCG in the liver, followed by entry into systemic circulation (Blachier and Andriamihaja, 2022). Free forms of *p*-cresol, pCS, and pCG circulate and are excreted in the urine, while the bulk of these compounds are reversibly bound to albumin.

The sulfotransferase enzyme, SULT1A1, catalyzes the transfer of a sulfonate group (SO_3_^-^) to *p*-cresol, resulting in the pCS product. This transfer is dependent upon an adequate sulfur pool in the liver. As with sulfation, glucuronidation (e.g., UGT1A6) converts the lipophilic *p*-cresol into the more water-soluble and excretable pCG. This conjugation is driven by UDP-glucuronosyltransferases (Rong and Kiang, 2020).

In circulating plasma, roughly 95% of *p*-cresol is bound to albumin in the healthy and in those with CKD (Deltombe et al., 2015). pCS is among the most concentrated and prevalent of all the circulating metabolites derived from the human gut microbiome, evidenced by high mean circulating values of 230 µM in uremic patients and 15 µM in healthy individuals (Vanholder et al., 2014).

### 1.3 Biological effects and relevance of *p*-cresol to human spaceflight

Gut microbial changes in humans have previously been reported in short- and long-duration space missions (Tesei et al., 2022; Tierney et al., 2024; Etlin et al., 2024; Kim et al., 2024; Overbey et al., 2024). The related *p*-cresol dynamics are of specific importance because of *p*-cresol’s low molecular mass of 108.1 Da, its high permeability (penetrates both the blood brain barrier and the intestinal barrier), and its widespread impact on the clinical phenotype (Stachulski et al., 2023). An overview of the *p*-cresol biological effects is presented below.

#### 1.3.1 Impact of *p*-cresol on the heart and circulatory system

*p*-Cresol elevation is of potential importance to cardiovascular health in space, since it has been associated with processes such as reactive oxygen species generation (e.g., superoxide), endothelial dysfunction, epithelial-to-mesenchymal transition, leukocyte–endothelial interaction, deterioration of cardiac cell functional capacity, expression of tissue factor in vascular smooth muscle cells, renal dysfunction, and insulin resistance (Vanholder et al., 2014). *p*-Cresol may exert some of its endothelial effects via activation of epidermal growth factor receptor (EGFR) signaling, which sequentially activates the intracellular signaling proteins annexin A1 and STAT3, leading to mobilization of matrix metalloproteinase MMP-2/9 and disruption to the integrity of the BBB. This effect can be reversed by pharmacological blockade of the EGFR receptor (Shah et al., 2024).

Since 2019, the issue of jugular vein stasis and thrombosis in spaceflight has gained considerable attention as a new space risk of concern. While the etiology is complex, the findings of Nemet et al. draws added attention to the role that *p*-cresol may or may not play as a covariate in thrombosis in space (Nemet et al., 2023). In their longitudinal study, *p*-cresyl sulfate and indoxyl sulfate were shown to predict 5-year mortality risks. After identifying the microbes and microbial enzymes involved in the generation of these uremic toxins, they set out to examine a series of preclinical models involving *p*-cresol and showed that pCS led to a thrombotic phenotype (evidenced by the rate of platelet clotting following carotid artery injury and the time to cessation of flow within the carotid artery).

#### 1.3.2 Impact of *p*-cresol on the brain and nervous system

Among its primary CNS effects, *p*-cresol is mechanistically associated with altered dopamine metabolism (Pascucci et al., 2020) via direct inhibition of dopamine-β-hydroxylase (DBH; (Vinithakumari et al., 2022). *p*-Cresol does so by forming covalent bonds with two different tyrosine positions of DBH (amino acids 216 and 357), resulting in irreversible inhibition of the enzyme and elevated dopamine levels (DeWolf et al., 1988; Umek et al., 2018). At cytosolic pH, dopamine is unstable and excess is readily converted to dopamine-o-quinones and aminochrome, which generate considerable oxidative stress (Muñoz et al., 2012).

Not unexpectedly, then, *p*-cresol has been shown to be elevated in the cerebrospinal fluid (Sankowski et al., 2020) and serum (Cirstea et al., 2020) in Parkinson’s (PD) disease compared with controls. In a study of 250 with Parkinson’s disease, Lin et al. found that pCG and pCS were significantly elevated in PD compared with controls and that elevated pCG was correlated with motor symptom severity (Chen et al., 2023). Sankowski et al. showed through targeted metabolomics that levels of pCS in PD were roughly eight times higher in CSF than in plasma (Sankowski et al., 2020). Whether this concentration disparity exists in healthy humans is unknown at this time. *p*-Cresol is known to increase BBB permeability, which aligns with the known increased BBB permeability found in PD (Gray and Woulfe, 2015; Lau et al., 2024).

In a recent profile of 700 metabolites and concurrent assessment of global cognitive function, *p*-cresol was among the top three compounds differentiating the low from the high cognition group (Palacios et al., 2019). Bertarini et al. have extensively reviewed the neurotoxic effects of *p*-cresol, as outlined in **Table 2**.

**Table 2:** Adverse effects of *p*-cresol and its metabolites on nervous system function.

Function	Adverse effects of *p*-cresol
Oxidative Stress	Increased reactive oxygen species (ROS), GSH depletion, and imbalance between pro- and antioxidant systems
Mitochondria	Impaired Krebs cycle, structural mitochondrial damage, and energy imbalance;
Inflammation	Damage to neurons and oligodendrocytes: activation of inflammatory signaling pathways (e.g., NF-κB, JNK), elevated IL-1β levels, and reduced neuronal survival markers
Synapse	Reduced synaptic density, impaired expression of key synaptic proteins, and dendritic remodeling deficits
Receptor Activity	Altered NMDA receptor or subunit composition, receptor mislocalization, and reduced Na+/K+-ATPase activity
Neurotransmitter	Dopamine accumulation due to DBH inhibition, reduced 5-HT (serotonin)
Neurotrophins	Reduced BDNF; reduced phosphorylation of CREB, a transcription factor crucial for regulating genes involved in synaptic plasticity, neurogenesis, and neuronal survival.
Immune	Inhibition of Th1 immune responses, decreased IFN-γ, and shift toward a Th2 cytokine profile
Microglia	Microglial dysfunction and impaired brain immune responses: suppression of pro-inflammatory cytokine release (TNF-α, IL-6) and inhibition of ADAM10/17 activity (a Zn^2+^-dependent transmembrane and secreted metalloprotease superfamily, so-called “molecular scissors.”)

**Function** (adapted from (Bertarini et al., 2025).

#### 1.3.3 Impact of *p*-cresol on bone and the skeletal system

Beyond its CNS effects, *p*-cresol appears to have a mechanistic association with impaired bone metabolism, which has the potential to adversely affect one of the key risks of human spaceflight—bone loss. In a series of *in vitro* studies, Kamprom et al. set out to examine the impact of *p*-cresol on the osteogenic differentiation of bone marrow-derived mesenchymal stem cells under different *p*-cresol concentrations from 0 to 14 days of differentiation into osteoblasts. *p*-Cresol was found to 1) downregulate the expression of a key osteogenic-associated gene; *COL1A1* which encodes for collagen type I protein, 2) reduce alkaline phosphatase in a dose-dependent manner, 3) dose-dependently reduce calcium deposition of MSC-osteoblastic cells, and 4) markedly induce MSC-osteoblastic cells to undergo senescence during osteogenic differentiation (in association with activation of the *p21* gene) (Kamprom et al., 2021). In patients with kidney disease, there exists a robust association between the highest *p*-cresol tertile and bone fracture risk (Chang et al., 2021). These convergent findings may hold important insights for those working to remedy the challenge of bone loss in space.

#### 1.3.4 Impact of *p*-cresol on the kidneys and renal system

The kidney is one of the most extensively studied organs with respect to the impact of *p*-cresol on Earth, with these kidney dynamics being seen as a two-way street (Huang et al., 2025a). Fundamentally, *p*-cresol can lead to direct renal toxicity, acute kidney injury, and chronic kidney disease (CKD). As kidney function deteriorates, uremic toxins accumulate, whereby processing of toxins such as *p*-cresol can become impaired and inflict further damage to the kidneys and circulatory system, thus establishing a viscous cycle.

As concentrations of compounds such as *p*-cresol rise, patterns of acute kidney injury emerge including their associated processes of increased oxidative stress, inflammation, apoptosis, and fibrosis, resulting in compromise to the different tissue components of the blood vessels, heart, and kidney. Clinically, pCS has been associated with progression of CKD and with all-cause mortality (Wu et al., 2011; Lin et al., 2014).

At a functional level, elevated *p*-cresol has been shown to be correlated with reduced eGFR in the healthy as well as in those with chronic kidney disease. Barrios et al. found that pCS, indoxyl-sulfate, and phenylacetylglutamine were inversely associated with eGFR in a large UK population cohort (UKTwins; n = 4439) and that microbiome changes associated with these markers are early markers of renal function decline (Barrios et al., 2015).

Siew et al. recently examined 20 independent human and rodent spaceflight missions/simulations that had sufficient data or samples from which they could generate a novel data set (Siew et al., 2024). They applied what they described as a ‘pan-omics approach incorporating anatomical and physiological parameters.’ They concluded that galactic cosmic rays (GCR) and microgravity likely converge to drive renal functional and structural remodeling, including an elevated risk to nephrolithiasis due to calcium loss from bone. They also showed that acute exposure to GCR causes acute and chronic tubular epithelial and vascular damage, which may be progressive and some of which may be irreversible. *p*-Cresol dynamics may be among the lesser studied contributors to these changes.

#### 1.3.5 Impact of *p*-cresol on hepatic function and drug metabolism

*p*-Cresol may also negatively impact the biotransformation of endogenous metabolites and selected medications that require sulfur conjugation (**Figure 3**). As noted, the hepatic metabolism of *p*-cresol leads to the formation of pCS and pCG. Persistent elevation of pCS over time can deplete the hepatic sulfur pool and reduce the available sulfur groups needed to metabolize drugs (acetaminophen, levothyroxine, amoxicillin, morphine, hydrocortisone, etc.; Isvoran et al., 2022), endogenous compounds (estrogens, androgens, dopamine, norepinephrine, serotonin, bile acids, thyroid hormones, etc.; Glatt et al., 2001; Ebmeier and Anderson, 2004; Bairam et al., 2018), and xenobiotics.

**Figure 3:**
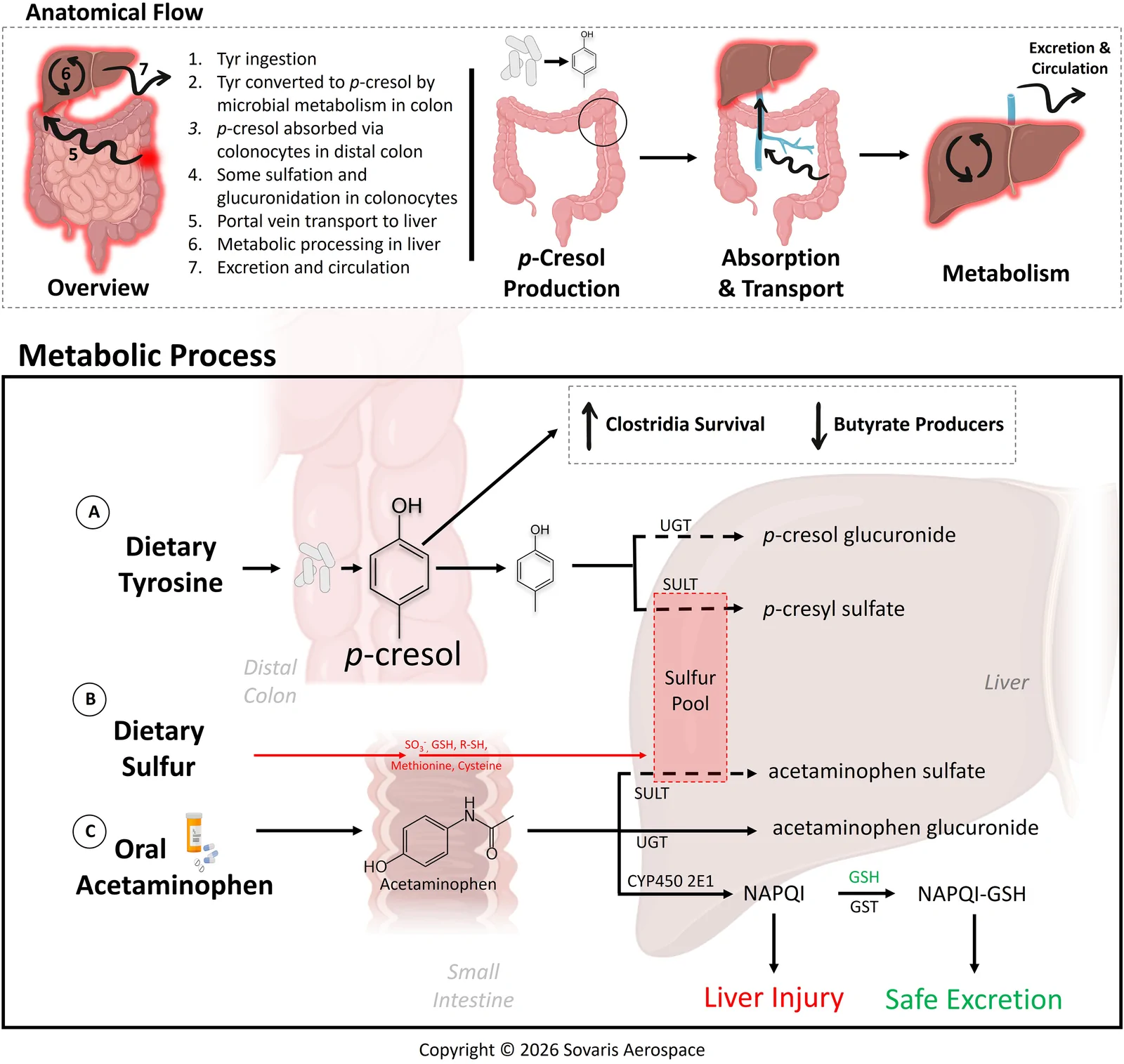
*p*-Cresol formation and its impact on molecular networks in astronauts. The figure shows three processes with intersecting relationships: **(A)** Dietary tyrosine is converted by colonic microbiota to *p*-cresol. *p*-Cresol is absorbed into hepatic circulation, where it is metabolized by UGT into pCG and by SULT into pCS. *p*-Cresol sulfation utilizes and requires a sufficient hepatic sulfur pool. *p*-Cresol that remains in the colon inhibits butyrate producing microbes, while facilitating survival of clostridial species (*p*-cresol producers); **(B)** Dietary sulfur groups (sulfur pool) are provided in the form of SO3-, glutathione, cysteine, methionine, and others. These replenish the hepatic sulfur pool needed to remove drugs, endogenous compounds, and xenobiotics. The presence of *p*-cresol can deplete the hepatic sulfur pool needed for the safe metabolism of drugs like acetaminophen in space; **(C)** Oral acetaminophen is absorbed through the small intestine and transported to the liver where it is metabolized by UGT into acetaminophen glucuronide and by SULT into acetaminophen sulfate. The latter process directly competes with *p*-cresol for SULT activity and for sulfur molecules. Up to 15% of acetaminophen is also metabolized by CYP4502E1 into NAPQI. In the presence of adequate glutathione and active GST, NAPQI is rendered harmless and excreted (as NAPQI-GSH). In the absence of adequate glutathione, however, NAPQI is hepatotoxic; Tyr: Tyrosine; UGT: UDP-glucuronosyltransferase; GSH-glutathione; SULT-sulfotransferase; NAPQI-N-acetyl-p-benzoquinone imine; RSH-generic sulfhydryl compound; GST-glutathione-S-transferase.

For instance, Clayton et al. administered 1 gram of acetaminophen to humans, while assessing urinary pCS, acetaminophen sulfate, and acetaminophen glucuronide. They observed that individuals with high pre-dose urinary levels of pCS had a lower post-acetaminophen urinary ratio of acetaminophen sulfate to acetaminophen glucuronide, suggesting that the declining hepatic sulfur pool (due to *p*-cresol competition for hepatic sulfur groups) caused a shift in acetaminophen metabolism away from sulfation and toward glucuronidation (Clayton et al., 2006).

A depleted sulfur pool is of further concern because inadequate phase II sulfur conjugation of acetaminophen (following CYP4502E1 biotransformation) can result in formation of the hepatotoxin *N*-acetyl-*p*-benzoquinone imine (NAPQI). Unconjugated NAPQI binds to proteins and subcellular structures and induces rapid cell death and necrosis, which can lead to liver failure. Ordinarily, an adequate supply of glutathione (GSH) enables formation of an NAPQI-GSH conjugate for safe excretion. However, *p*-cresol has been shown to lower GSH, which suggests the presence of *p*-cresol may render the formation of NAPQI more probable (Edamatsu et al., 2018).

#### 1.3.6 Impact of *p*-cresol on the gut microbiome and colonic health

As noted, one detrimental effect of *p*-cresol is the inhibition of butyrate producing microbes in the gut, the depletion of which can have an adverse impact on gastrointestinal mucosal integrity via depleted butyrate (Wong et al., 2014). At higher concentrations, *p*-cresol uncouples colonocyte cellular respiration and ATP synthesis, ultimately diminishing the ATP concentration within cells (Andriamihaja et al., 2015). Other GI effects are as noted previously.

*p*-Cresol has also been associated with the development of colon cancer (Diether and Willing, 2019; Zhang et al., 2025). Mechanistically, *p*-cresol elevation induces the upregulation of proinflammatory cytokines, such as IL-6 and further induces dysregulation of lipid metabolism (Li et al., 2015). Clinically, *p*-cresol levels have been shown to be significantly elevated in the feces of those with colon cancer when compared to healthy individuals (Alustiza et al., 2023). To this point, *p*-cresol has been proposed as a potential biomarker for the detection of pre-malignant lesions and colon cancer.

#### 1.3.7 Impact of *p*-cresol on skin

In the present study of 340 days in space, astronaut TW reported significant skin symptoms on return to Earth, which included: 1) highly sensitive skin, reacting to contact with surfaces, 2) hive-like rashes on areas of skin that came in contact with surfaces, such as sitting or lying down, and 3) sensation of skin burning when it touched anything (Law et al., 2020).

Flight surgeons have plausibly attributed his skin reactivity to the absence of skin contact over the long-duration mission. However, a question arises as to the possible role of elevated *p*-cresol as a potential variable for consideration in the genesis of these symptoms.

For instance, in one cohort of 320 CKD patients, 35% had clinically significant pruritus, and *p*-cresol sulfate was confirmed as an independent predictor of itch severity via multivariate logistic regression (Wang et al., 2016). *p*-Cresol has been shown to disturb keratinocyte differentiation (Iizuka et al., 2009). CKD and uremic patients show profound xerosis characterized by reduced skin hydration, increased stratum corneum desquamation, and markedly elevated transepidermal water loss (Wang et al., 2016). These changes correlate with impaired keratinocyte differentiation, reduced intercellular lipid content, and depleted skin glycerol — all downstream effects of *p*-cresol and related uremic toxins (Wang et al., 2016).

For the reasons cited above (including those highlighted in **Figure 4**), we extracted the plasma *p*-cresol features (pCS and pCG) derived from the untargeted metabolome during one year in space, examined its association with gut microbial community structure governing *p*-cresol and butyrate synthesis, genes responsible for gut microbial *p*-cresol and butyrate synthesis, genes associated with *p*-cresol biotransformation, and consider the implications for astronaut health, astronaut performance, and precision medicine applications on long-duration spaceflight.

**Figure 4:**
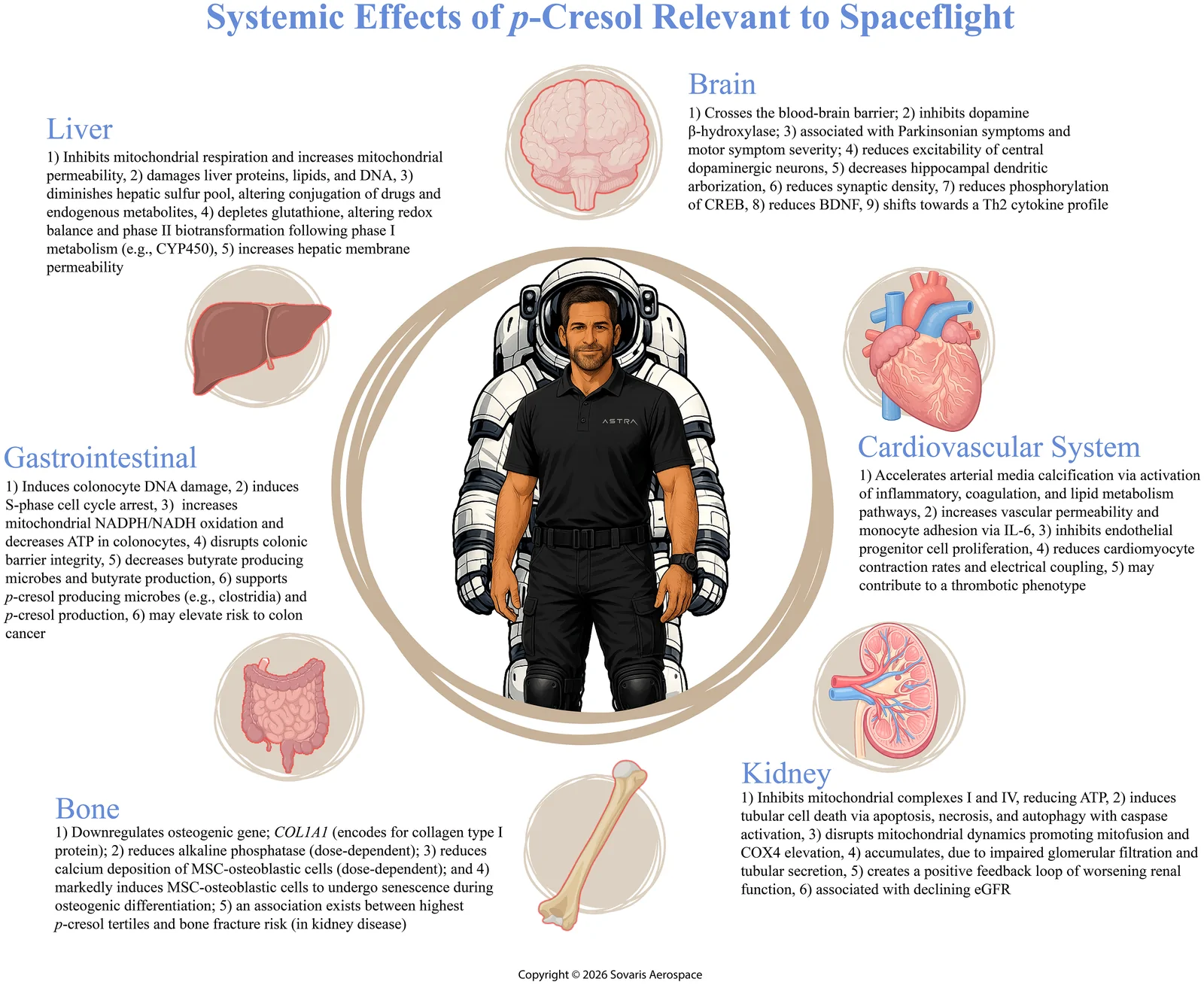
Systemic effects of *p*-cresol in humans.

## 2 Materials and methods

### 2.1 Study participants

A pair of monozygotic twins were studied for 25 months, during which one subject (Flight subject, TW) spent 340 days aboard the International Space Station (ISS) while his identical twin (Ground subject, HR) remained on Earth. Subjects were male and aged 50 at the beginning of the study. Both subjects had different amounts of spaceflight exposure prior to the study (Flight subject=180 days total, Ground subject=54 days total). Multiple blood samples were collected (N_flight_=19, N_ground_=12) from both subjects beginning approximately 6 months prior to the launch date, during the 340 days aboard the ISS and 6 months after return.

### 2.2 Sample collection

All of the freshly collected blood samples aboard the ISS from TW were returned on the Soyuz capsule. Therefore, samples derived from ISS habitation were confounded by ambient return to Earth. To correct for the effects of ambient return on gene expression, blood samples were collected from an unrelated individual, which were then exposed to conditions that simulated the fresh and ambient return collections. The fresh and ambient samples from the control individual were processed and sequenced with the same protocol used for the study subjects. Data generated from the control individual was used to model and correct the effects of ambient return, using ComBat or multivariate analyses in differential expression, as previously described (Garrett-Bakelman et al., 2019).

### 2.3 Untargeted plasma metabolomics

Plasma metabolites were profiled using a broad-spectrum LC-MS platform as previously described (Contrepois et al., 2015; Piening et al., 2018). Metabolites were extracted from plasma using a 1:1:1 acetone:acetonitrile:methanol solvent mixture, dried under nitrogen, and reconstituted in 1:1 methanol:water. Samples were analyzed by untargeted LC-MS using complementary HILIC and reverse-phase LC methods in both positive and negative ionization modes, with pooled samples used for MS/MS-based metabolite identification and repeated quality control injections throughout the run sequence.

Raw files were converted and processed in R using XCMS for feature detection, alignment, and quantification and CAMERA for annotation. Features from blanks, poor linear signals, and those present in fewer than 33% of samples were removed; missing values were imputed, signal drift was corrected by LOESS normalization using pooled QC samples, and metabolites were identified by matching retention time and fragmentation spectra to reference databases. Data were then log-transformed, median-scaled, and analyzed with a linear model, with false discovery rate adjustment applied to identify significant metabolites at adjusted p<0.05 (Garrett-Bakelman et al., 2019). The two *p*-cresol conjugates were extracted from the metabolomics dataset across all 25 HR/TW timepoints: *p*-cresol glucuronide and *p*-cresyl sulfate. Graphs of the untargeted plasma metabolomics data were made using Microsoft Excel in Microsoft Office 365.

### 2.4 Microbiome metagenomics

Stool samples were collected by swabbing within 2 hours of voiding using the Hardy Diagnostics Envirotrans™ Swab Rinse Kit and were stored at -80^∘^C until processing. Genomic DNA was extracted from 2mL of saline suspension using the BiOstic Bacteremia DNA Isolation Kit. DNA quality was assessed with a Qubit spectrophotometer and Agilent TapeStation 2200, followed by acoustic shearing with a Covaris S2. Sequencing libraries were prepared using the Accel-NGS 1S Plus DNA Library Kit, initially sequenced on an Illumina MiniSeq, then re-pooled based on read output and sequenced on an Illumina NextSeq500 using a high-output 300-cycle kit (Garrett-Bakelman et al., 2019).

Sequencing data were processed through two parallel taxonomic classification pipelines. In the Primary Pipeline, reads were profiled with MetaPhlAn2 (Truong et al., 2015), which identified 168 species across 22 fecal metagenome samples; relative-abundance estimates from this pipeline were used in the majority of downstream comparisons. In the Secondary Pipeline, reads were classified with KrakenHLL (Pockrandt et al., 2022), which produced species-level read counts for 641 species across 18 annotated samples. KrakenHLL counts were next used as input for DESeq2 differential abundance analysis (Love et al., 2014).

Outputs from both pipelines were cross-referenced against the curated list of known *p*-cresol and butyrate producers reported in Saito et al (Saito et al., 2018), identifying 12 matching *p*-cresol producers and 14 matching butyrate producers. KrakenHLL count data were filtered to features with at least 10 reads in at least 3 of 18 samples prior to DESeq2 analysis; 324 species passed this filtering threshold. Thirteen contrasts were computed for adjusted p-values: within-twin temporal comparisons (3 each for TW and HR), between-twin comparisons at each phase (3), interaction contrasts (3), and a pooled TW vs. HR comparison. Species reaching significance in DESeq2 that also appeared in the Saito et al. reference list (Saito et al., 2018) were flagged for further interpretation. All *p*-cresol and butyrate producers were aggregated to yield total producer abundances for group-level comparisons.

All abundance reporting (with the exception of *Clostridioides difficile* and *Olsenella uli*) was derived from the Primary Pipeline (MetaPhlAn2) and is reported as Relative Abundance in their respective Results section. Only *Clostridioides difficile* and *Olsenella uli* are reported as Count Normalized Abundance and are reported in section 3.2.1. as being derived from the Secondary Pipeline (KrakenHLL DESeq2).

Gut enzymes of interest were identified using HUMAnN2 UniRef90 gene families, then regrouped to EC numbers using the HUMAnN3 utility mapping, targeting six enzymes from the *p*-cresol and butyrate synthesis pathways.

### 2.5 Transcriptome preparation, sequencing, and analysis

Transcriptome preparation, sequencing, and analysis were conducted as published in *Garrett-Bakelman* et al (Garrett-Bakelman et al., 2019). Briefly, RNA-seq libraries were generated from purified and bulk-cell populations using two complementary approaches: ribosomal RNA depletion and poly(A) mRNA enrichment, following standard NEBNext directional library preparation protocols with matched RNA inputs, randomized indexing, and pooled sequencing to minimize batch effects. High-quality RNA was used for all samples, with protocol-specific fragmentation, cleanup, and PCR amplification conditions optimized for each library type. Universal Human Reference RNA with ERCC spike-ins was included as a control. Libraries were sequenced as pooled samples on the Illumina HiSeq 4000 platform with paired-end 150 x 150 reads.

Raw and processed sequence data were assessed with FastQC and MultiQC, adapter-trimmed using Trim Galore!, and aligned to the hg19 genome and Gencode v19 annotation, including ERCC spike-ins, using two-pass STAR alignment. Gene and transcript abundance were quantified with featureCounts and kallisto, respectively, and summarized at the gene level. Differential expression analysis was performed separately by cell type using DESeq2 on raw count data, with multivariate models incorporating subject, flight status, library preparation method, and ambient return effects. From this dataset, transcripts for *SULT1A1, SULT2A1, SULT1A3, SULT1A4, and CYP4502E1* were targeted due to their biological significance in *p*-cresol. Graphs of the transcriptomics data were made using R.

### 2.6 Dietary analysis

Dietary intake analysis was not conducted on this study, other than a limited food frequency questionnaire (FFQ) on TW only. This data was not considered suitable or dependable for follow up analysis. It also provided no capacity for comparison between HR and TW (as there was no FFQ for HR on Earth). Plasma tyrosine was also not assessed.

### 2.7 Nomenclature

For the purpose of clarity, the following nomenclature conventions were used. TW refers to the astronaut that entered space (aka, Flight), while HR refers to the astronaut remaining on Earth (aka, Ground). This labeling convention was used to maintain consistency with prior NASA Twins Study publications describing the astronauts. The terms Pre-flight, Inflight, and Post-flight refer to mission stages that correspond to 1) Earth-based sample collection of TW and HR before flight, 2) ISS-based sample collection (TW) in low Earth orbit (and the parallel sample collection of HR on Earth), and 3) Earth-based sample collection from TW and HR, upon TW return to Earth and its follow up, respectively.

Also, we report selected results using the term Interaction, which is a comparison in the difference of differences (Love et al., 2014). Interaction captures whether the change in microbiome composition or abundance over time in TW is significantly greater or lesser than the change observed in HR over the same period. In other words, it isolates the microbial response uniquely attributable to the spaceflight environment above and beyond any time-related shifts shared by both subjects.

## 3 Results

### 3.1 Plasma *p*-cresyl sulfate and *p*-cresol glucuronide

Plasma pCS and pCG rose during spaceflight and returned to normal levels upon return to Earth (**Table 3**, **Figure 5**). pCS and pCG were also elevated in the space condition when compared with the ground-based control. Significance was observed for pCG when comparing Post-flight vs. Inflight (adj p = 0.016). pCS values were significantly altered Post-flight vs. Inflight (adj p = 0.002) and Post-flight vs. Late-flight (adj p = 0.037). pCS values for Early-flight vs. Pre-flight approached significance (adj p = 0.080).

**Table 3:** Adjusted *p*-value comparisons for untargeted metabolomics of pCG and pCS.

Comparison	*p*-Cresol glucuronide	*p*-Cresyl sulfate
TWvsHR	0.199	0.555
InflightVsGround	0.324	0.101
PostflightVsGround	0.593	0.593
Time	0.611	0.830
TimeSpentInISS	0.915	0.767
TimeAfterReturn	0.485	0.310
InflightVsPre-flight	0.324	0.101
Post-flightVsPre-flight	0.593	0.593
Post-flightVsInflight	0.016*	0.002*
Early-FlightVsPre-flight	0.201	0.080**
Late-FlightVsPre-flight	0.684	0.349
Post-flightVsLate-Flight	0.172	0.037*
Late-FlightVsEarly-Flight	0.761	0.761

* p < 0.05.

** p approaching significance is in reference to the p-value set at the significance level of p = 0.05.

**Figure 5:**
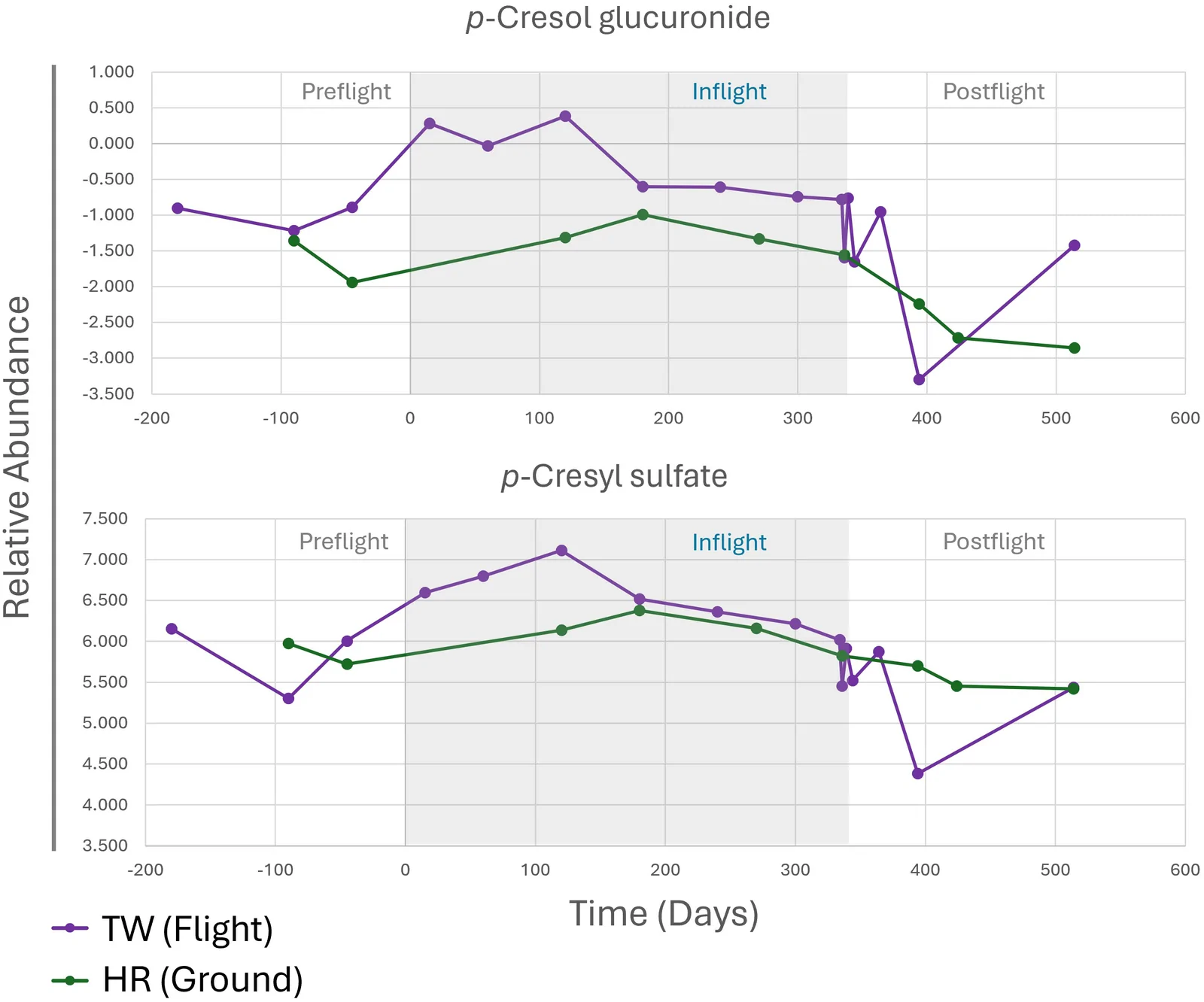
Change in plasma *p*-cresol metabolites during one year in space (units: median-normalized log intensity; relative).

### 3.2 Abundance of fecal *p*-cresol producing microbes during one year in space

#### 3.2.1 Count-normalized abundance of *Clostridioides difficile* and *Olsenella uli*

Results derived from the Secondary Pipeline for *Clostridioides difficile* and *Olsenella uli* are reported separately here (**Figure 6**), based on the notable status of these two microbes as being among the top four *p*-cresol producers (major *p*-cresol producer >100 µM), as reported by Saito e al (Saito et al., 2018). Of note, we observed an increase in count-normalized abundance for *Clostridioides difficile* in TW (Flight) vs. HR (Ground) for Post-flight, Inflight, and Overall. (adj p = 0.014, adj p = 0.0006, and adj p = 2.02x10¯^5^, respectively). Concomitantly, we observed a significant increase in count-normalized abundance for *Olsenella uli* in TW vs. HR for Post-flight, Inflight, and Overall (adj p = 0.0045, adj p = 0.00043, adj p = 0.0016, respectively), including Interaction for Inflight vs. Pre-flight (adj p = 0.028).

**Figure 6:**
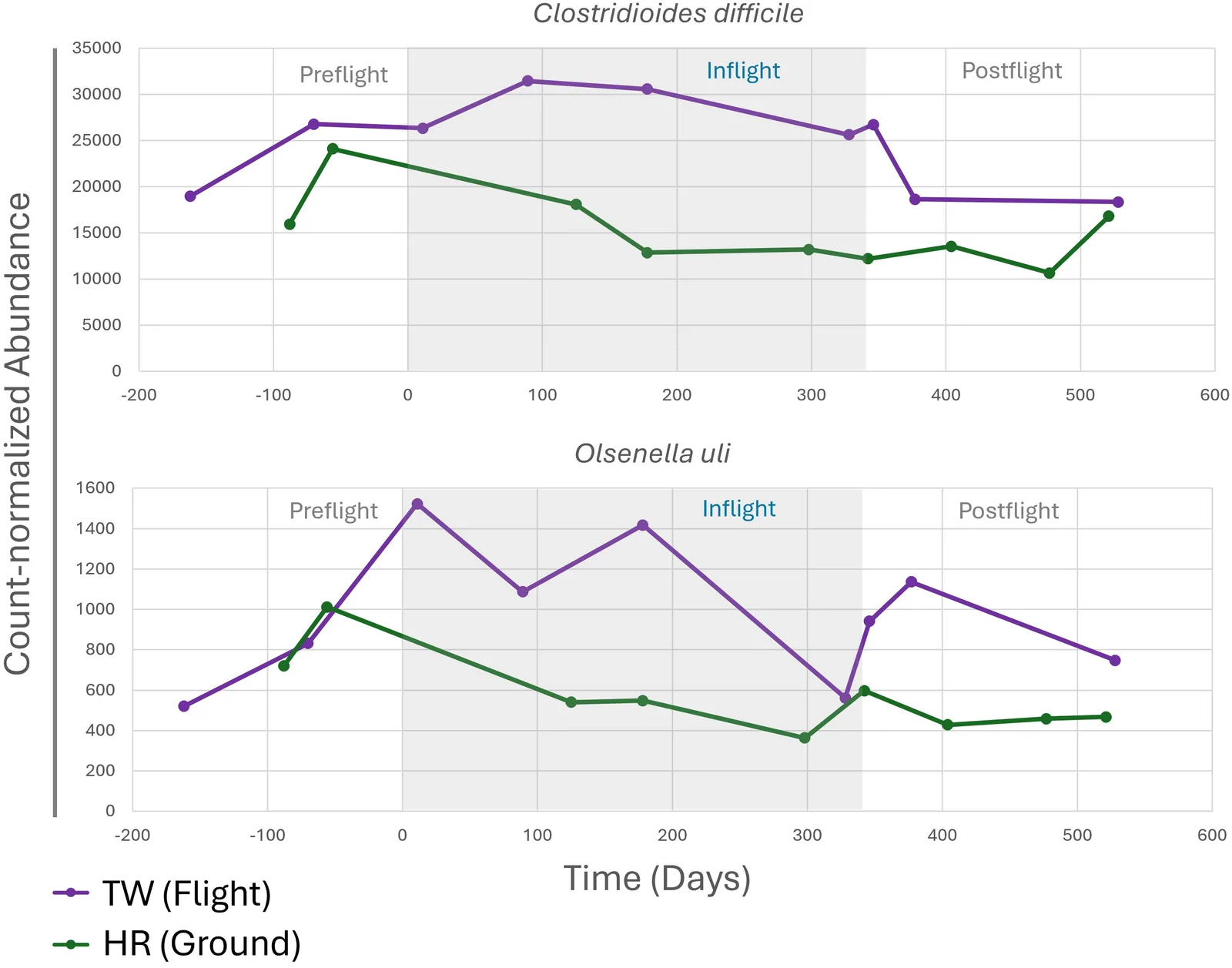
Count-normalized abundance of *Clostridioides difficile* and *Olsenella uli*.

#### 3.2.2 Relative abundance of other fecal *p*-cresol producing microbes during one year in space

Across the general *p*-cresol-producing taxa detected in the Primary Pipeline (**Figure 7**), TW exhibited several clear temporal shifts over the mission, although the magnitude and direction of change differed by organism. Among the most visually notable changes were those observed *for Bacteroides uniformis, Clostridium leptum, Clostridium innocuum, Eubacterium rectale*, and *Bacteroides caccae*. *B. uniformis* was highest Pre-flight, declined during Inflight, and then increased again near the Inflight-to-Post-flight transition. *B. caccae* remained low for most of the mission but showed a sharp transient spike near the end of Inflight or beginning of Post-flight. This visual pattern was supported by significant differences for TW Post-flight vs. Inflight (adj p = 0.0024) and TW vs. HR Post-flight (adj p = 0.0013).

**Figure 7:**
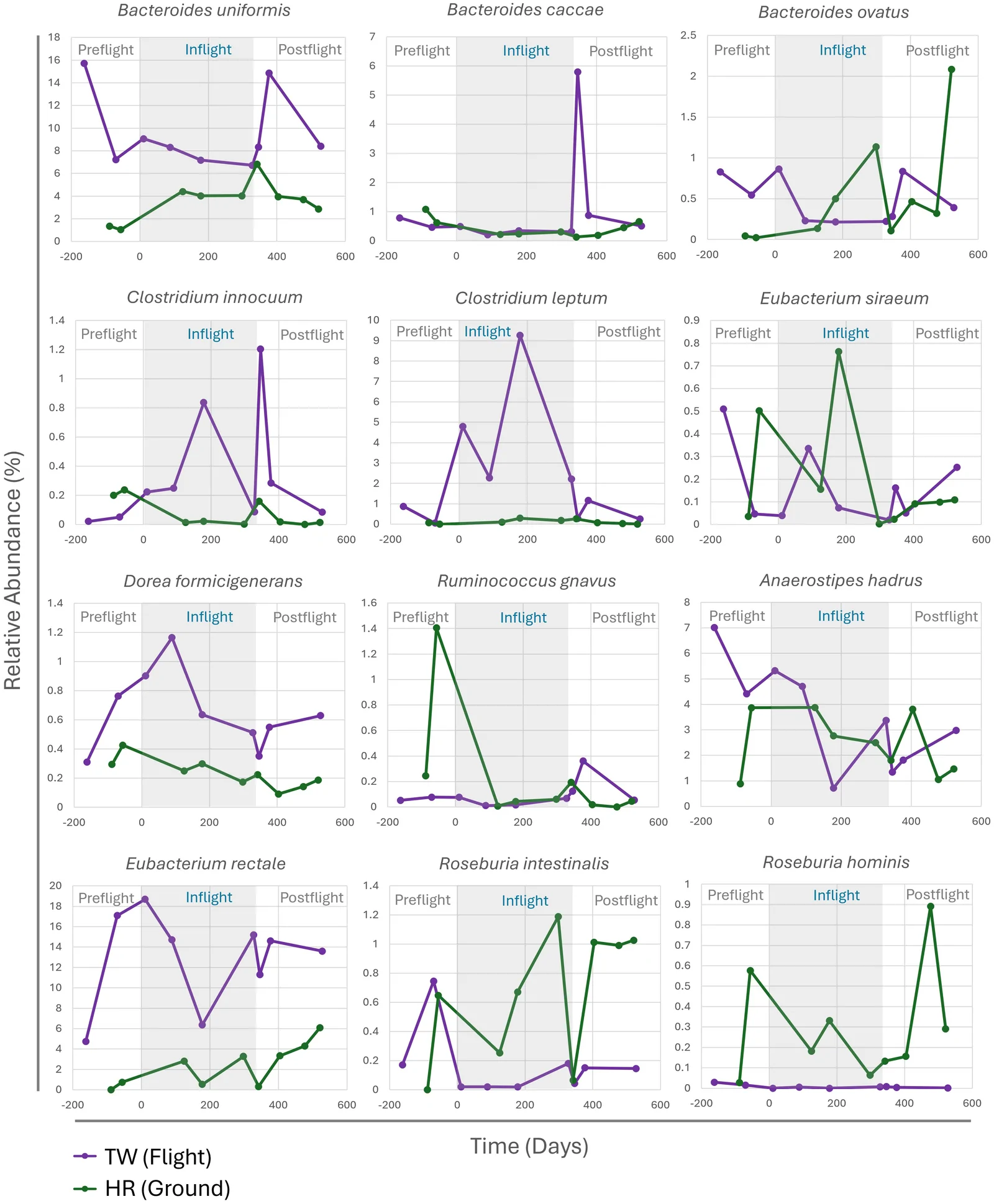
Change in relative abundance of individual fecal *p*-cresol producing microbes during one year in space. Results were derived from examining the presence of known *p*-cresol producing species outlined in **Table 1** with *p*-cresol producing species detected in the present study cohort.

In contrast, *Bacteroides ovatus* remained low overall in TW and did not show a clear sustained Inflight enrichment, although it was one of the taxa with significant associations, including TW vs. HR Pre-flight (p = 0.0039), interaction Pre-flight vs. Post-flight (adj p = 0.0080), interaction Inflight vs. Pre-flight (adj p = 0.0009), ground Post-flight vs. Pre-flight (adj p = 0.0003), and ground Inflight vs. Pre-flight (adj p = 0.0001). *Dorea formicigenerans* increased from Pre-flight to early Inflight and then declined later but did not show significant associations.

Among the Firmicutes-associated taxa, *C. leptum* showed one of the clearest Inflight increases in TW, rising from low Pre-flight abundance to a pronounced Inflight peak before declining thereafter. *C. innocuum* also increased during Inflight and reached a late Inflight peak, followed by reduction after landing. *Eubacterium siraeum* trended downward overall from Pre-flight into later timepoints in TW despite some Inflight fluctuation, while *E. rectale* remained relatively abundant throughout the mission and displayed marked variability across Inflight, with an early elevation, a mid-Inflight decline, and subsequent rebound. By contrast, *Ruminococcus gnavus*, *Roseburia intestinalis*, and *Roseburia hominis* showed little evidence of consistent Inflight elevation in TW, while *Anaerostipes hadrus* was higher Pre-flight and lower during Inflight with partial later recovery. None of these taxa showed statistically significant associations in the modeled comparisons.

The most pronounced statistical pattern among *p*-cresol producers was observed for *Acidaminococcus fermentans*, which demonstrated significant associations for TW vs. HR Pre-flight (adj p = 1.1x10^-123^), TW vs. HR overall (adj p = 1.55x10^-55^), HR Post-flight vs. Pre-flight (adj p = 2.1x10^-256^), TW Inflight vs. Pre-flight (adj p = 2.7x10^-146^), interaction Post-flight vs. Pre-flight (adj p = 1.13x10^-83^), and interaction Inflight vs. Pre-flight (adj p = 1.8x10^-76^). Although not among the most visually emphasized taxa in the longitudinal plots, these results indicate a strong differential temporal pattern between TW and HR.

In HR, several taxa also varied over time, although these changes were generally distinct from the dominant flight-associated patterns. *B. uniformis* remained well below TW and increased modestly during Inflight before tapering later, whereas *B. caccae* and *B. ovatus* stayed low overall aside from a late Post-flight rise in *B. ovatus*. *D. formicigenerans* varied only modestly in HR. Among the Firmicutes-associated taxa, *C. leptum* and *C. innocuum* remained low and relatively stable in HR, contrasting with their more pronounced Inflight increases in TW. *E. siraeum* showed a distinct transient Inflight peak in HR, and *E. rectale* rose gradually over time despite remaining below TW at most time points. *R. gnavus* displayed an isolated high Pre-flight value in HR followed by low abundance thereafter, while *R. intestinalis* and *R. hominis* fluctuated more in HR than in TW. *A. hadrus* remained moderately abundant throughout the ground series, with variability but without the pronounced Pre-flight-high to Inflight-low shift seen in TW. All other taxa comparisons were not significant.

### 3.3 Abundance of fecal butyrate producing microbes

For butyrate-producing taxa derived from the Primary Pipeline (**Figure 8**), significant associations were observed only for *Faecalibacterium prausnitzii*. Specifically, *F. prausnitzii* was significant for TW vs. HR Pre-flight (adj p = 0.0164), the interaction of Post-flight vs. Pre-flight (adj p = 0.0080), and HR Post-flight vs. Pre-flight (adj p = 0.0271). All other taxa comparisons were not significant. Consistent with these statistical findings, *F. prausnitzii* also showed clear temporal variation by visual inspection. Specifically, in TW, it decreased from Pre-flight levels, rose during Inflight to a late Inflight peak, and then dropped sharply after landing, whereas in HR it increased gradually across the mission and reached a Post-flight high.

**Figure 8:**
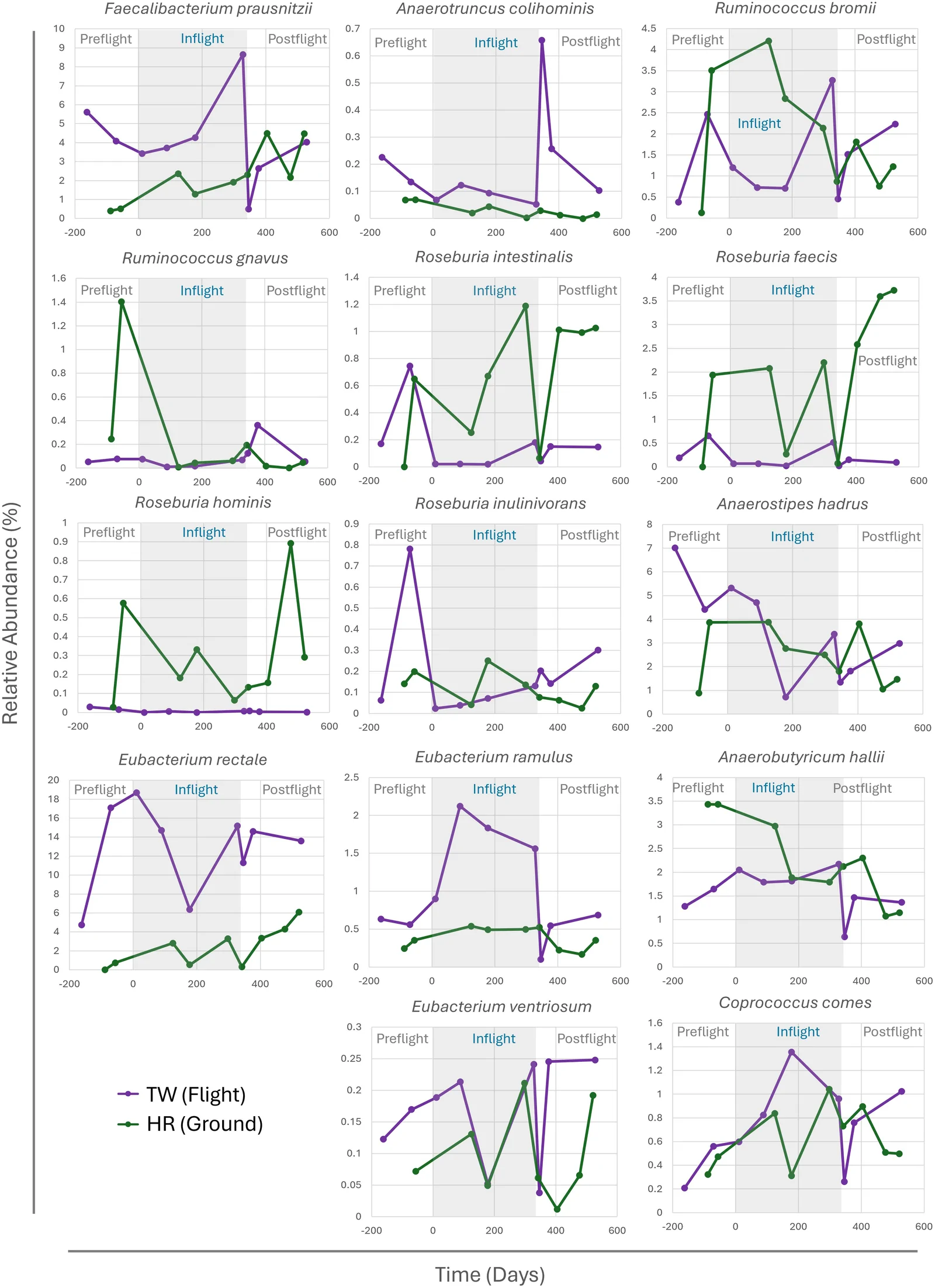
Change in relative abundance of individual fecal butyrate producing microbes during one year in space. Results were derived from correlating known butyrate producing species outlined in **Table 1** with butyrate producing species detected in the present study cohort.

Several other butyrate-producing taxa showed visually apparent temporal shifts across the mission, although these were not statistically significant and should therefore be interpreted descriptively rather than inferentially. In TW, *Anaerotruncus colihominis* remained low for much of the time series but displayed a pronounced late Inflight spike followed by Post-flight reduction. *Anaerostipes hadrus* was relatively abundant Pre-flight, declined substantially during Inflight, and showed only partial recovery afterward. *Eubacterium rectale* remained abundant throughout but fluctuated considerably, dropping from high Pre-flight levels during Inflight before rebounding later.

More moderate or transient changes were observed for *Eubacterium ramulus*, which increased from Pre-flight into Inflight and then declined; *Ruminococcus bromii*, which decreased during Inflight and rebounded afterward; and *Coprococcus comes*, which increased and peaked during flight before declining after landing. In contrast, *Anaerobutyricum hallii* appeared comparatively stable, and several additional taxa, including *Roseburia intestinalis*, *Roseburia faecis*, *Roseburia hominis*, *Roseburia inulinivorans*, and *Ruminococcus gnavus*, remained at low relative abundance with only limited fluctuation.

In HR, several butyrate-producing taxa also varied over time, in some cases more than in TW. *F. prausnitzii* rose gradually across the mission, while *A. colihominis* remained very low throughout. *R. bromii* was higher during Pre-flight and early Inflight before declining later, and *R. gnavus* showed a marked Pre-flight elevation followed by near-baseline values. *R. intestinalis*, *R. faecis*, and *R. hominis* were more variable in HR, with notable later increases or spikes, whereas *R. inulinivorans* remained low overall. *A. hadrus* stayed moderately abundant throughout the ground series and did not mirror the pronounced Inflight decline seen in TW. *E. rectale* increased progressively over time in HR while remaining below TW at most time points. *E. ramulus* remained relatively stable at low-to-moderate abundance, *A. hallii* showed a mild overall decline, and *Coprococcus comes* fluctuated modestly without a sharp isolated excursion.

Aggregate change in total relative abundance of fecal *p*-cresol and butyrate producing microbes during one year in space are reported in **Figure 9**.

**Figure 9:**
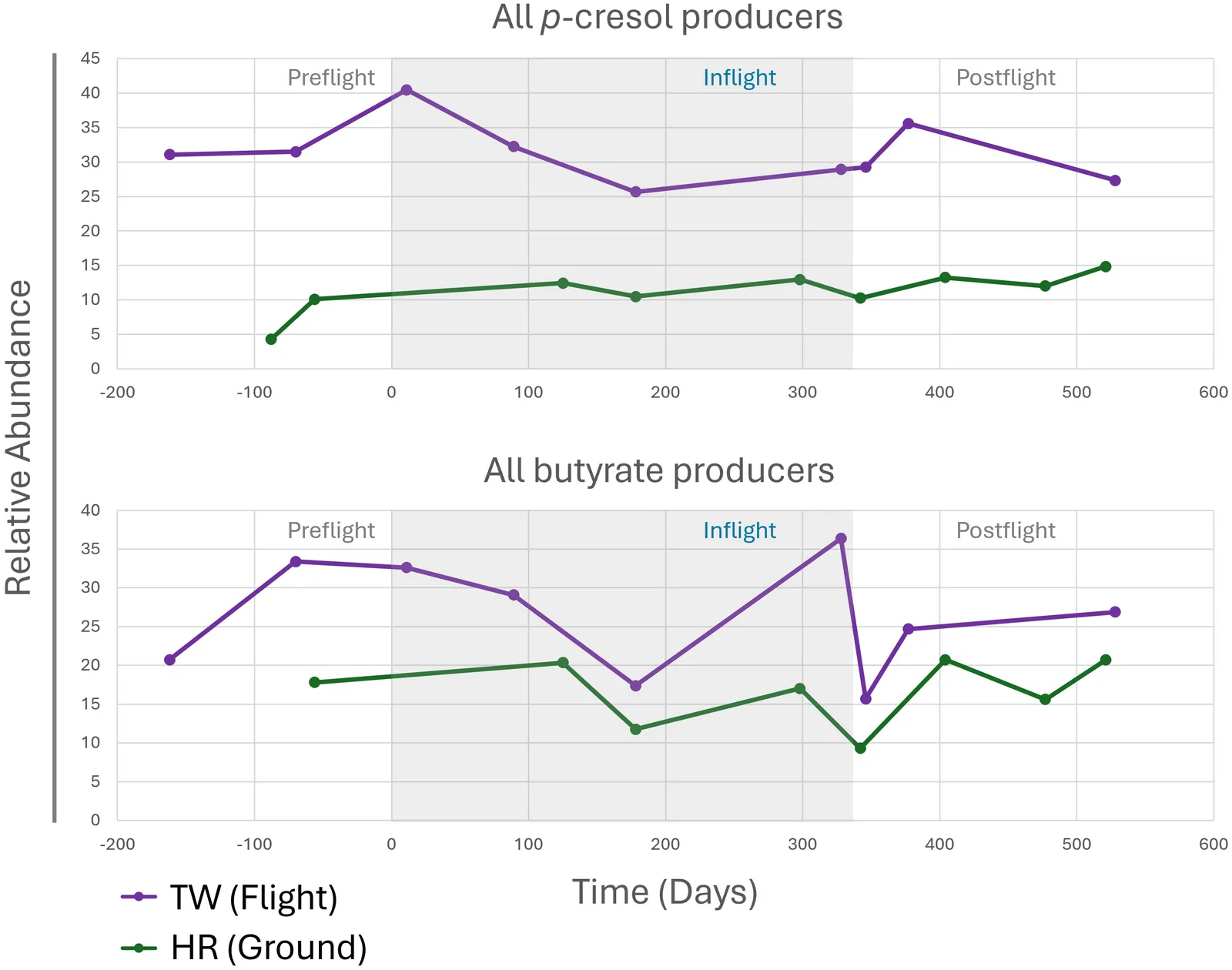
Aggregate change in total relative abundance of fecal *p*-cresol and butyrate producing microbes during one year in space.

### 3.4 Abundance of gut microbial genes associated with *p*-cresol formation

To further understand the pathways and networks associated with *p*-cresol formation from tyrosine, we investigated the fecal DNA associated with four target enzymes relevant to *p*-cresol metabolism across 21 samples. Included were urease, uricase, tyrosine ammonia lyase, and hydroxyphenylacetate decarboxylase (Saito et al., 2018). Only the DNA for urease was detected using the untargeted methods in the study (**Figure 10**).

**Figure 10:**
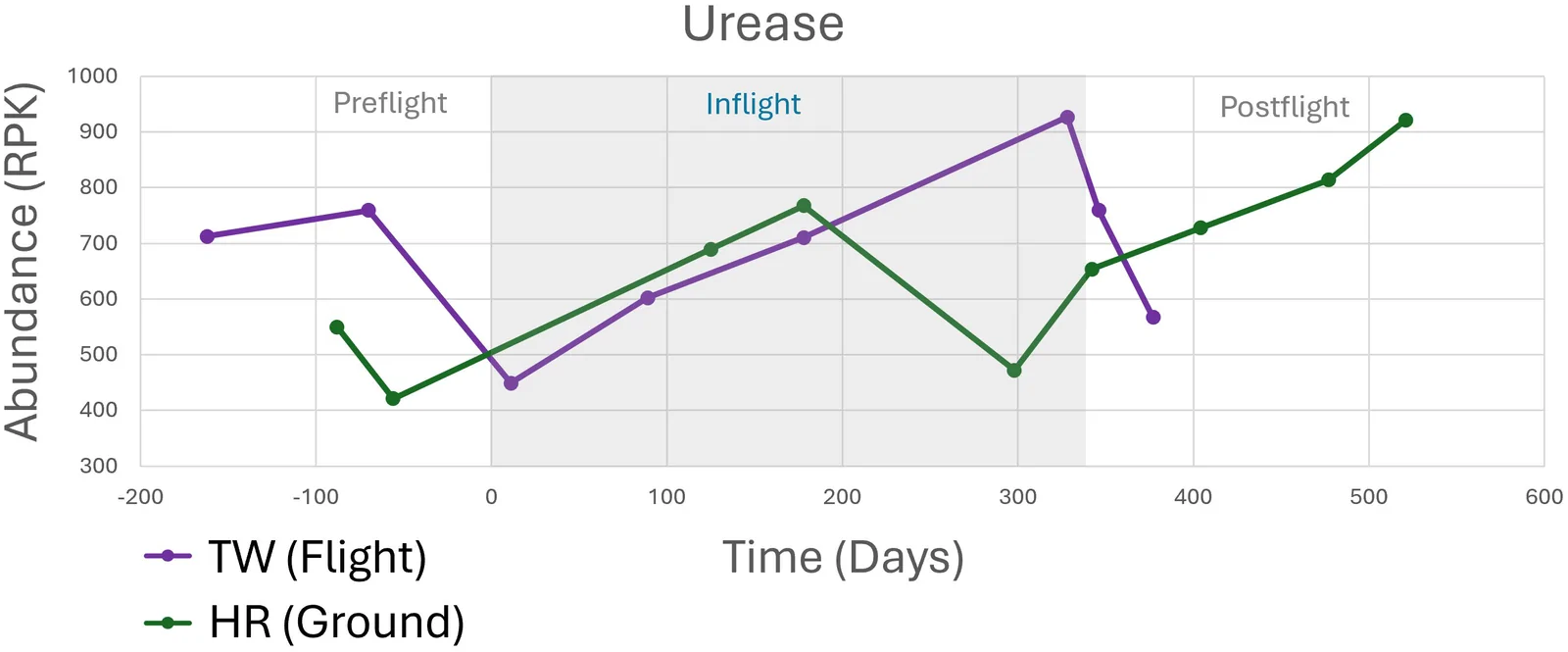
Change in RPK abundance of gut microbial urease genes associated with *p*-cresol formation.* Uricase (1.7.3.3), Tyrosine Ammonia Lyase (ThiH; 4.3.1.23), and Hydroxyphenylacetate decarboxylase (Hpd: 4.1.1.83) were not detected. E.C. numbers represent the enzyme associated with their respective gene.

### 3.5 Abundance of gut microbial phosphotransbutyrylase and butyrate kinase genes associated with butyrate formation

To further understand the pathways and networks associated with butyrate formation, we investigated the fecal DNA associated with two target enzymes relevant to butyrate metabolism across 21 samples (**Figure 11**). This included features for phosphotransbutyrylase and butyrate kinase. .

**Figure 11:**
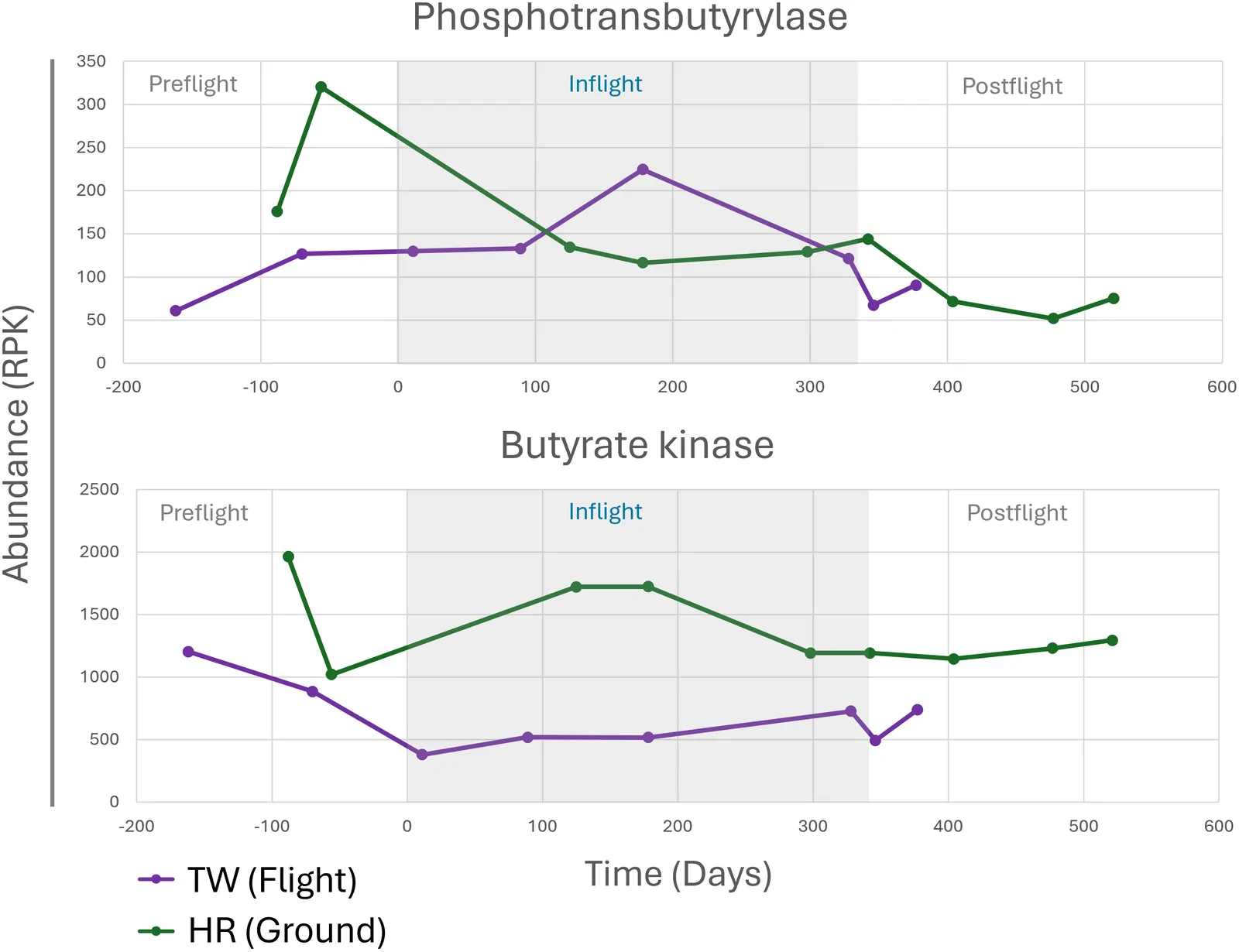
Change in RPK abundance of gut microbial phosphotransbutyrylase and butyrate kinase genes associated with butyrate formation. Phosphotransbutyrylase (2.3.1.19) and Butyrate Kinase (2.7.2.7). E.C. numbers represent the enzyme associated with their respective gene.

### 3.6 White blood cell *cytochrome P4502E1* RNA transcripts

No consistent patterns of change were observed in *CYP4502E1* in the numerous white blood cell types reported here. This would suggest that *CYP450 2E1* is not significantly impacted by 340 days in space. However, white blood cell *CYP4502E1* transcripts rose roughly 2-fold at specific timepoints and in specific cell types during spaceflight, specifically in the CPT fraction (PBMC). The Cell Preparation Tube (CPT) is an evacuated, sterile blood collection tube containing buffered sodium citrate or sodium heparin anticoagulant, liquid density medium, and an inert gel barrier. Since *hepatic CYP4502E1* was not measured, it cannot be determined from the present data whether elevated *p*-cresol was accompanied by alteration of hepatic *CYP4502E1* activity (**Supplementary Figure 1**).

### 3.7 White blood cell sulfotransferase RNA transcripts

No significant changes in sulfotransferase RNA (*SULT1A1*, *SULT1A3*, *SULT1B1*, *SULT1E1*, and *SULT2A1*) were detected in the numerous white blood cell types reported here. This would suggest that WBC sulfotransferases are not significantly impacted by 340 days in space. Since *hepatic* sulfotransferases were not measured, it cannot be determined from the present data whether elevated *p*-cresol was accompanied by alteration of hepatic sulfotransferase activity (**Supplementary Figure 2**).

### 3.8 White blood cell glutathione *S*-transferase RNA transcripts

No significant changes in glutathione-*S*-transferase P1 (*GSTP1*) RNA were detected in the numerous white blood cell types reported here. This would suggest that *GSTP1* is not significantly impacted by 340 days in space. However, since *hepatic GSTP1* was not measured, it cannot be determined from the present data whether elevated *p*-cresol was accompanied by alteration of hepatic *GSTP1* activity (**Supplementary Figure 3**).

### 3.9 Body mass and nutrition

TW’s energy intake was about 66% of predicted requirements, and beyond the nominal vitamin D supplements provided to all astronauts, TW did not report taking vitamin or mineral supplements during the mission. In addition, TW lost 7% of his body mass during flight, whereas HR gained ~4% of his body mass over the course of the study.

## 4 Discussion/conclusion

We report herein on the first evidence that pCS and pCG are elevated during long-duration human spaceflight (Garrett-Bakelman et al., 2019; Schmidt et al., 2020). In alignment with this result, we observed a commensurate increase in the count-normalized abundance of *Clostridioides difficile* and *Olsenella uli*, two of the most efficient producers of *p*-cresol in humans. Collectively, the results may reflect a changing dynamic of the gut microbiome in human spaceflight and a previously unknown effect of spaceflight regarding the converging factors that govern *p*-cresol production in humans. This may have widespread effects on astronaut molecular networks, physiological systems, and the clinical phenotype.

To better understand the context of our findings, it is useful to restate that the *p*-cresol elevation we observed was measured in relative units. Absolute concentrations and their relationship to clinically significant thresholds await quantitative confirmation in future studies.

The attention to countermeasures here serves two purposes. First, any follow up *observational* hypothesis testing requires proper experimental controls and annotation. This necessitates an understanding of many of the potential influences (variables) summarized in the countermeasure section below. Second, follow up *interventional* research requires knowledge of potential countermeasure candidates, so that candidate interventions can be thoughtfully selected or that countermeasure candidates not selected for study can be controlled or annotated. Thus, the countermeasure discussion provides investigators engaged in follow up research a framework from which future experimental design can be informed. A summary of the experimental findings and related considerations is below.

### 4.1 *p*-Cresyl sulfate vs. *p*-cresol glucuronide

We observed that the relative Inflight increase in pCG reported here is greater than pCS over time. In fact, pCS declines from its initial elevation, as the mission progresses. Terrestrially, it has been well established that both pathways of *p*-cresol conjugation (sulfation and glucuronidation) are active in humans. However, because *p*-cresol consumes limiting hepatic sulfur groups (through conjugation and subsequent elimination of the *p*-cresol-sulfur conjugate; pCS), sustained *p*-cresol exposure over time can reduce available hepatic sulfur groups needed for further conjugation, resulting in a shunting of *p*-cresol metabolism to the glucuronidation pathway (thus resulting in sustained *p*-cresol glucuronide production, as observed here).

In theory, the declining pCS with time reported herein may indicate a decline in the sulfur pool over time in space, though an actual decline in *p*-cresol cannot be ruled out. This determination awaits further examination on future missions.

#### 4.1.1 *p*-Cresyl sulfate and *p*-cresol glucuronide post-flight trajectory

There are two patterns in the Post-flight data that warrant attention. First, the downward Post-flight trendline for pCS and pCG were similar. Second, there was a repeating pattern of rising and falling *p*-cresol where the pCS and pCG levels across the 8 Post-flight collection points temporally mirror one another (**Figure 5**). Although we lack the longitudinal sampling density and direct tissue-compartment measurements to definitively explain this variability, the pharmacokinetic properties of pCS derived from Earth-based research offer a mechanistically coherent interpretive framework for future hypothesis testing.

*p*-Cresol has a volume of distribution approximately four times greater than creatinine, indicating extensive sequestration in peripheral tissue compartments beyond the plasma compartment (Lesaffer et al., 2003). Approximately 95% of circulating pCS is reversibly bound to albumin, with only the free fraction available for renal filtration or tissue exchange at any given moment (Deltombe et al., 2015). These properties together mean that when the source of *p*-cresol production is perturbed — whether by dialysis removing circulating toxin or, as in the present case, by post-landing normalization of gut flora and dietary context — plasma levels may initially decline as the free fraction is cleared, but tissue-sequestered and albumin-bound pCS continues to redistribute back into the free plasma compartment, generating a secondary elevation that lags the source perturbation by hours to days.

In the dialysis literature, this phenomenon has been quantified. Preliminary data from Lesaffer et al. demonstrated that up to 50% of the intra-dialytic decrease in *p*-cresol concentration was neutralized by post-dialytic rebound within hours of treatment cessation (Lessafer et al., 2003). Mathematical modeling of protein-bound uremic toxins kinetics (PBUT) using a three-compartment patient model has further formalized the interplay between plasma, tissue, and protein-bound reservoirs in governing the time-course of pCS following perturbation (Maheshwari et al., 2017).

Applied to the post-flight context, individual variation in three factors may plausibly account for the two observed patterns: (1) the magnitude of tissue compartment burden accumulated over 340 days, which governs the rate and duration of redistribution back into plasma following the source perturbation of landing; (2) residual hepatic sulfur pool capacity and SULT1A1 functional status post-flight, which determines how rapidly pCS conjugation and renal clearance can be re-established; and (3) the trajectory of gut flora recovery following return to Earth, including whether abundance of major *p*-cresol producers such *as Clostridioides difficile* and *Olsenella uli* normalizes immediately upon landing or persists into the post-flight period. Given the documented capacity of *C. difficile* to suppress competing commensal taxa via *p*-cresol-mediated inhibition (Passmore et al., 2018), a prolonged post-landing persistence of the dysbiotic state is biologically plausible and would sustain *p*-cresol production even after the microgravity and radiation environment has been removed.

Taken together, it is reasonable to consider that these pharmacokinetic and microbiological considerations suggest that the two Post-flight patterns observed in this study are not anomalous findings requiring explanation by unique clinical events but are instead predictable consequences of well-characterized PBUT distribution dynamics interacting with individual variation in conjugation capacity and microbiome recovery rate. Future studies incorporating higher-frequency post-flight sampling, direct fecal and plasma *p*-cresol and butyrate quantification, and SULT1A1 functional assays would allow formal testing of this hypothesis.

### 4.2 Microbes and genes associated with *p*-cresol and butyrate production

If one presumes that the elevation in plasma *p*-cresol metabolites is a product of colonic microbial metabolism, it would also be expected that some pattern of gut microbial change consistent with that dynamic would be evident. To that end, the finding of an increase in the count-normalized abundance of *Clostridioides difficile* and *Olsenella uli* as primary *p*-cresol producers is consistent with that mechanistic association.

However, it bears noting that there is a possibility the count-normalized abundance (as opposed to relative abundance) for these two species represents a false positive and that any interpretation of these results should be approached with caution. Nevertheless, we felt compelled to report these results of the count-normalized abundance based on the following rationale: 1) we observed two (rather than only one) high *p*-cresol producing species of significance in the raw reads; 2) these two species exist within the top four of known *p*-cresol producers; 3) the change in these two species correspond with the plasma *p*-cresol trajectory in space; 4) several comparisons of significance were observed for each of pCS, pCG, *C. diff*., and *O. uli*. To further this rationale, both *C. diff.* and *O. uli* comfortably pass the threshold of 10 reads in all 18 samples (*C. diff.* min 10,666 reads; *O. uli* min 363 reads), so neither was borderline.

With regard to fecal RPK gene abundance associated with *p*-cresol production, there was insufficient data from which to draw conclusions, given that urease was the only gene of interest detected (out of four) and there was no pattern of significance related to change in urease in space. Consistent with the mechanism of *p*-cresol suppression of butyrate producing microbes, the RPK abundance of butyrate kinase (a primary gene associated with butyrate production) was lower for TW vs. HR Inflight and across the entire observation period. In contrast, RPK abundance for phosphotransbutyrylase showed no consistent pattern. It is important to note that fecal butyrate data was not available, which would have been necessary to more fully characterize the gut butyrate dynamics in space.

### 4.3 Cytochrome P4502E1

While CYP4502E1 does not engage in direct metabolism of *p*-cresol, CYP2E1 is actively involved in the metabolism of drugs like acetaminophen, which is impacted by *p*-cresol both in *p*-cresol’s adverse effect on the sulfur pool (i.e., on sulfation via sulfotransferase) and on glutathione synthesis (impacting phase II GSH conjugation of drugs like acetaminophen). We observed that white blood cell *CYP4502E1* transcripts rose roughly 2-fold at specific timepoints and in certain cell types during spaceflight, specifically in the CPT fraction (PBMC).

The transient upregulation of *CYP4502E1* in space may reflect a variable response to the spaceflight condition or ingestion of a medication used during the mission that was not disclosed. Consistent with the former, in rat liver, three gray (Gy) of γ-irradiation (^60^Co) has been shown to trigger a 3.6-fold increase in *CYP4502E1* mRNA at 24 h, in contrast to no effect on the expression of *CYP4501A2* and *CYP4503A*. If the transient elevation of *CYP4502E1* expression reflects a spaceflight-specific response, further investigation will be needed to elucidate the nature of that response. It is also important to note that the hepatic *CYP4502E1* isoform was not measured in this study. This would have given a more accurate reflection of the primary site of *p*-cresol biotransformation.

### 4.4 Sulfotransferase and glutathione-*S*-transferase

Sulfotransferase enzymes are important in space, because this enzyme is responsible for the sulfur conjugation of compounds such as *p*-cresol, as well as drugs such as acetaminophen and a wide range of endogenous compounds. The finding of no significant change in white blood cell SULT does not rule out an effect of space, since most transsulfuration takes place in the liver. Nevertheless, the finding of minimal change in WBC adds to the body of literature in space.

The behavior of glutathione-*S*-transferase genes in relation to *p*-cresol in space is of added importance, since low GST1 in the presence of drugs such as acetaminophen can result in low glutathione conjugation of acetaminophen and formation of hepatotoxins, such as NAPQI. As with SULT, white blood cells do not provide an indication of the behavior of the hepatic pool of GST1, which is where the preponderance of this enzymatic activity takes place.

Understanding the impact of changes to the sulfur pool in space will become central to developing appropriate countermeasures. For instance, Kurosawa et al. found a decrease in hepatic sulfur antioxidants (glutathione, cysteine, taurine, ergothioneine, etc.) in mice during a 30-day mission on the ISS (Kurosawa et al., 2021). Moreover, GSH decreased, while its oxidized form GSSG (glutathione disulfide) increased, suggesting the GSH ability to carry out its conjugation and antioxidant roles in space is compromised in mice. The untargeted nature of the present study did not provide for the appropriate controls needed for quantitative assessment of GSH and its related compounds.

### 4.5 *p*-Cresol countermeasure candidates in human spaceflight

Should the findings reported herein be replicated using fully quantitative measures in a cohort of sufficient size, the question will arise as to what might be reasonable countermeasures for consideration at some future point in the advancement of human space missions. An alternate view one might entertain is that of precision medicine. In this latter case, one might assume that *p*-cresol presents a signal of potential importance and that, having measured the associated molecular networks, one could proceed with the application of precision medicine today, i.e., a tailored approach to each astronaut that would be applied to current missions. In either case, it is useful to outline a set of plausible countermeasure candidates either as targets for future research or as candidates for clinical application today (or both). .

A brief overview of countermeasure candidates for consideration in the study of elevated *p*-cresol in space as summarized below might include 1) nutrient modulation, 2) dietary changes, 3) prebiotics, 4) probiotics, 5) drugs, and 6) fecal biobanking and fecal microbiota transplant (**Table 4**). Pre-mission assessment of selected provisional biomarkers coupled with personalized intervention also warrant consideration.

**Table 4:** Countermeasure candidates for *p*-cresol mitigation in astronauts.

Class	Agent(s)	Primary mechanism
Nutrients	Glutathione (GSH) N-acetylcysteine (NAC)	Amino acids, tripeptides, and other compounds that replenish depleted compounds impacted by *p*-cresol; nutrients that support direct conjugation of *p*-cresol; nutrients that support drug and endogenous metabolite conjugation and processing.
Prebiotics	Inulin Fructooligosaccharides Galactooligosaccharides Xylooligosaccharides Human milk oligosaccharides Resistant starches Polyphenols Lactulose	Fermentable substrates, oral supplements, and foods that colonic microbes utilize as substrates for metabolism, growth, colony expansion, competition, and survival. Used to enhance the growth of intestinal microbial species that compete with *p*-cresol producers. A key output of prebiotic fermentation is the production of short chain fatty acids such as butyrate, acetate, and propionate.
Probiotics	Beneficial bacteria (Lactobacilli, Bifidobacteria, etc.) Fermented foods containing beneficial bacteria	Oral dietary supplements containing beneficial microbes for delivery to the colon, which are intended to establish residence and act via competitive inhibition against *p*-cresol producing microbes (and improve microbial diversity). Fermented foods of a wide variety also provide beneficial bacteria.
Drugs	AST-120 Acarbose (Precose, Prandase) Inhibitors of HpdBCA decarboxylase Antibiotics	AST-120 is an oral charcoal compound used to reduce the levels of certain uremic toxins via adsorption, such as *p*-cresol in people with chronic kidney disease. Acarbose is an alpha-glucosidase inhibitor that acts on carbohydrate in the gut. Antibiotics may be indicated in certain cases where pathogenic organisms such as *C. difficile* require more aggressive management, though collateral impact on microbial community structure must be considered.
FMT (Fecal Microbiota Transplant)	Autologous or non-autologous donor feces	In principle, FMT is similar to oral probiotics in that the FMT is intended to establish a healthy colonic microbial community structure. FMT reduces plasma *p*-cresol levels in recipients, coupled with increased urinary *p*-cresol levels (urinary excretion) (Zhou et al., 2023). Consider pre-mission biobanking of astronaut fecal samples for later use in FMT.

#### 4.5.1 Nutrient candidates for *p*-cresol modulation

At the substrate level, dietary restriction (tyrosine or phenylalanine restriction) has been proposed as one means to effectively reduce *p*-cresol levels, as it reduces tyrosine intake from dietary protein sources and removes the crucial substrate for microbial conversion to *p*-cresol (Fernandes et al., 2020). For instance, in one study comparing vegetarians to omnivores consuming an unrestricted diet for one month, *p*-cresyl sulfate excretion was 62% lower in vegetarians, which coincided with a fiber intake 69% higher and protein intake 25% lower than the *ad libitum* omnivores (Patel et al., 2012).

While dietary tyrosine restriction may seem intuitive, an associated reduction in total protein intake would further reduce the lower protein and caloric intake already common in space missions. Lowering protein intake (or tyrosine specifically) would also reduce the precursor available for neurotransmitter synthesis (e.g., dopamine, norepinephrine, epinephrine) and for thyroid hormone. Lowered protein would further reduce the intake of sulfur-bearing amino acids (e.g., L-cysteine and L-methionine) needed for *p*-cresol (and other compounds) sulfur conjugation and would reduce intake of branched chain amino acids needed for muscle protein synthesis (e.g., L-leucine).

At the endpoint level, attention to the sulfur pool should be among the lead considerations in addressing elevated *p*-cresol. *N*-acetylcysteine (NAC) is the accepted clinical means to treat acetaminophen poisoning in the emergency room setting, as it is known to reliably and rapidly replete the hepatic sulfur pool needed for acetaminophen sulfation (SULT1) and phase II glutathione conjugation (GST1) following CYP4502E1 biotransformation.

A parallel role of GSH also cannot be overlooked. As noted above, *p*-cresol directly inhibits dopamine-β-hydroxylase, leading to formation of dopamine oxidation products such as dopamine-*o*-quinones (DOQ) in brain structures like the substantia nigra. A critical function of GSH in the brain is the conjugation of unstable dopamine oxidation products into stable dopamine-*o*-quinone-GSH conjugates (DOQ-GSH) for safe elimination (catalyzed by GST-M2–2 and potentially amplifying the important role of GST in space; Baez et al., 1997; Zhou and Lim, 2010). However, this further depletes GSH stores, as the DOQ-GSH conjugate (and therefore GSH) is removed for elimination from the body.

It bears noting that when *p*-cresol triggers an oxidative stress response, a concomitant adaptive increase has been observed in the expression of γ-glutamylcysteine synthetase, which is the rate-limiting enzyme for glutathione synthesis (Edamatsu et al., 2018). This is the mechanism by which the body adapts to adverse oxidative conditions. However, GSH synthesis can only proceed in the presence of the sulfur amino acid cysteine, which is notably rate limiting. Once again, this demonstrates the importance of an optimized sulfur pool.

Overall, this DOQ-GSH phenomenon argues for careful attention to GSH status in the presence of gut microbial *p*-cresol production. While NAC is known to replenish the sulfur pool, it is also known to restore glutathione synthesis and protect the liver against NAPQI formation when acetaminophen is ingested (as GSH is a tripeptide of cysteine, glycine, and glutamic acid; Ben-Shachar et al., 2012). Oral or intravenous glutathione can also be considered as a pre-mission countermeasure. One could argue that provision of NAC or GSH is among the most reliable prophylactic measures to ensure adequate sulfur groups in the *p*-cresol exposure context, regardless of other steps taken.

#### 4.5.2 Dietary considerations for *p*-cresol modulation

A general consideration to address *p*-cresol in space is to prevent its formation via dietary modification of gut microbial communities using fermentable complex carbohydrate substrates. It bears noting, however, that much of the literature on modulation of *p*-cresol levels derives from the treatment of chronic kidney disease, which sometimes includes those on dialysis. These notably contain the confounding factors of CKD, its treatment, and its comorbidities. At the same time, the significant impact of dietary countermeasures and the considerable reduction in levels of *p*-cresol in selected studies provides insight to mechanisms that may be of value in spaceflight.

##### 5.5.2.1 Prebiotic considerations for *p*-cresol modulation

Prebiotics are dietary substrates that distribute to the colon where they are acted upon by colonic microbes. These can take the form of high molecular weight polymers (soluble and insoluble fibers) that are consumed in gram amounts daily (e.g., resistant starches from potatoes) or polyphenolic compounds that are more commonly consumed in the hundreds of milligrams daily (e.g., polyphenolics from berries and botanicals (Huang et al., 2025b).

Resistant starch is a carbohydrate that resists digestion in the small intestine. Resistant starch has recently been shown over 8 weeks (20–25 g/day) to significantly lower *p*-cresol in humans (Khosroshahi et al., 2019). In Wistar rats on a tyrosine-rich diet, resistant starch was shown to lower urinary *p*-cresol levels to that of controls (Chen et al., 2016).

In a 12-week feeding study, adult participants consumed 1) a control diet, 2) muffins with 10g/day pea hull fiber (4 weeks), and 3) muffins with 10g/day pea hull fiber and 15g/day inulin (6 weeks). Total fiber intake was 16.6 g/day, 26.5 g/day, and 34.5 g/day respectively. Providing pea hull fiber and inulin reduced serum *p*-cresol by 24% and by 37% in those with the greatest compliance (compliance being tied to greater inulin intake; Salmean et al., 2015).

Gondalia et al. engaged 80 participants in a randomized-controlled, double-blind trial using various whole and refined forms of wheat and resistant starch. High-amylose wheat is high in resistant starch and is known to modify enteric microbial populations. They showed that 4-week consumption of refined low-amylose wheat increased *p*-cresol producing bacterial abundance (i.e., clostridium; Peptostreptococcaceae family) and increased fecal *p*-cresol concentration. In contrast, *p*-cresol levels were reduced by refined high-amylose wheat ingestion, whole high-amylose wheat ingestion, and refined low-amylose wheat ingestion (Gondalia et al., 2022). Importantly, consumption of refined high-amylose wheat also resulted in a 38% greater fecal butyrate excretion, greater excretion of total short-chain fatty acids (SCFA), and increased fecal abundance of SCFA-producing bacteria (i.e., *Roseburia inulinivorans, Faecalibacterium* spp) when compared with those ingesting refined low-amylose wheat who experienced a 20% reduction in butyrate excretion.

In a cross-sectional study of adults with diabetes, the protein-fiber index was associated with total serum pCS (p = 0.005), independent of sex, diabetes, and eGFR. Interestingly, no significant association was found between dietary protein and *p*-cresyl sulfate levels (Rossi et al., 2015).

Selected mechanisms by which prebiotics modify gut health and the *p*-cresol dynamic, include but are not limited to: 1) facilitate growth (via fermentation) of beneficial bacteria such as *Bifidobacteria* and *Lactobacilli*, 2) support butyrate producers and production of short chain fatty acids such as butyrate, acetate, and propionate, 3) reduction of colonic pH, which deters pathogenic microbes, 4) anti-inflammatory actions, 5) support mucosal integrity via butyrate fueling colonocytes, 6) support mucosal integrity by enhancing tight junctions and reducing translocation (microbes, endotoxin, metabolites), 7) stimulate mucin production to support mucosal integrity, 8) facilitate microbial diversity, 9) stimulate antimicrobial peptide formation, and 10) direct inhibition of pathogens such as C. difficile (Davani-Davari et al., 2019; Ali et al., 2025; Yoo et al., 2024).

##### 5.5.2.2 Probiotic considerations for *p*-cresol modulation

Probiotics are defined as live microorganisms that, when administered in adequate amounts, confer a health benefit on the host. Synbiotics are merely a combination of prebiotics and probiotics. In one study of healthy volunteers, a synbiotic treatment, consisting of the probiotics *B. breve Yakult*, *L. casei Shirota* and the prebiotic oligofructose-enriched inulin, reduced urinary *p*-cresol (a surrogate marker for colonic *p*-cresol) and promoted the growth of bifidobacteria (De Preter et al., 2007). In a study by Guida et al, participants with elevated *p*-cresol and kidney disease were provided a synbiotic consisting of lactobacillus and bifidobacterial species, plus fructooligosaccharides, inulin (6.6g/day), and tapioca. They showed a 40% reduction in *p*-cresol over 30 days (Guida et al., 2014).

Candeliere et al. studied the degradation and formation kinetics of *p*-cresol using a method of resting gut microbial cells spiked with *p*-cresol to explore the ability of gut microbial species to biotransform *p*-cresol *in vitro* (Candeliere et al., 2022). The most active strains in *p*-cresol bioconversion were *Bifodobacterium longum* supsb. *longum*, *Lacticaseibacillus rhamnosus*, and *Lactiplantibacillus plantarum*. Though the precise mechanism of this bioconversion is unknown, such findings provide a basis for future examination of *p*-cresol modulation in humans.

#### 4.5.3 Drug considerations for *p*-cresol modulation

At this time, there is a limited repertoire of drugs to modulate *p*-cresol in humans. The primary drugs in use today are adsorptive in nature. For instance, AST-120 is an oral charcoal compound used to reduce the levels of certain uremic toxins, such as *p*-cresol, in patients with chronic kidney disease (Dhungana et al., 2025).

Acarbose is an anti-diabetic drug used to treat pre-diabetes and type 2 diabetes mellitus. It works by inhibiting alpha-glucosidase, which is an intestinal enzyme that liberates glucose from carbohydrate polymers (a so-called starch blocker). In a single study, Evenepoel et al. showed that Acarbose lowered *p*-cresol in one cohort of 9 adults when used for 3 weeks (Evenepoel et al., 2006).

An experimental drug approach to reduction of *p*-cresol employs the use of enzyme inhibitors at the gut level. Specifically, HpdBCA decarboxylase inhibitors are under investigation, which reduce *p*-cresol production in the gut and diminish the ability of clostridial species to compete with strains such as *Escherichia coli.* In one study, 4-Hydroxyphenylacetamide reduced *p*-cresol production by 54.9 ± 13.5%, while 4-Hydroxyphenylacetonitrile reduced *p*-cresol production by 99.0 ± 0.4% (Harrison et al., 2023).

#### 4.5.4 An overview of *p*-cresol modulator efficacy

An in-depth review of the efficacy of *p*-cresol modulators is not possible here. However, Dhungana et al. have recently addressed this topic with an analysis of 59 studies, including a total of 2593 study participants (Dhungana et al., 2025). A unique feature of this review is that it systematically evaluated interventions based on pharmacodynamics (effects of pCS reduction on clinical and laboratory outcomes) and pharmacokinetics (i.e., time dependency, dose dependency, effects on unbound vs bound pCS). Of the 59 studies, 27 demonstrated significant pCS reductions in patients. Note that there is considerable heterogeneity between the studies reviewed. However, these studies do provide a basis upon which future research and clinical decision support can be built.

In general, the concept of combination therapy or prophylaxis in response to the *p*-cresol phenomenon warrants consideration in research, prevention, and clinical care. For instance, a combination that gives attention to 1) dietary protein and amino acid intake (e.g., tyrosine), 2) gut microbial carbohydrate substrates (e.g., prebiotics), 3) live microbes (e.g., probiotics), 4) adsorbents (e.g., compounds such as AST-120 that adsorb and remove *p*-cresol from the gut), 5) antibiotics, and 6) hepatic conjugation agents that ensure sufficient hepatic sulfur groups for *p*-cresol conjugation in the liver and subsequent safe elimination (e.g., NAC, glutathione) may be of aggregate value.

It bears noting that any countermeasure consideration warrants attention to the fact that space food consists of irradiated, freeze-dried, or heat-stabilized products that may contain altered components (e.g., GSH, fatty acids, etc.) or inactivated components (e.g., probiotics) that might reduce their efficacy. In addition, the radiation of space may further degrade active components. These are among the fundamental challenges of human spaceflight in general.

### 4.6 Limitations and future directions

Several limitations of the present study warrant consideration, many of which are common challenges in human spaceflight. First, the small number of subjects (N = 2) is severely limiting and the large number of variables (analytes) renders such high-dimensional data prone to overfitting. One method to address this limitation is through numerous serial measures of the Pre-flight, Inflight, and Post-flight conditions, which was the approach used in this study. Second, the cohort of males is limiting on the generalizability of these results to females. Nonetheless, the sex-matched cohort favors homogeneity, which is valuable in small N studies involving high-dimensional data.

Third, the genomic data reported herein does not contain astronaut SNP, indel, CNV, and structural genomic elements for reasons of confidentiality. In particular, SNP variants of *CYP450*, *SULT*, *UGT*, *GST*, and others would have been additionally informative, as their impact on phase I and II biotransformation have been relatively well characterized. Thus, this analysis provides some insight into changes in molecular functional elements in the space environment, but it does not include an examination of genomic elements that might alter human risk in the space environment.

Fourth, the absence of hepatic transcripts of drug metabolizing enzymes remains and will remain a limitation until such difficult samples are obtained. Fifth, *p*-cresol elevation may be related to decreased GI transit time or alterations in renal clearance, which were not examined in this investigation (Gabriele et al., 2016; Roager et al., 2016).

Sixth, pCS and pCG levels in this study were determined using relative quantification, which limits our ability to infer whether they would translate into absolute concentrations of clinical significance. Greater attention to fully quantitative measurement of *p*-cresol (and its metabolites; plasma, urine, stool) and associated measures (e.g., fecal butyrate and short chain fatty acids) warrant consideration in future spaceflight research. Future analysis should also include another uremic toxin, indoxyl sulfate, which is a high concentration metabolite derived from gut microbial metabolism of dietary L-tryptophan.

Seventh, only four fecal samples were obtained in flight, which were not time-synchronized with blood, urine, and other sample collection. This frequency is limiting in general, which also limited direct temporal comparisons with blood values reported here. Eighth, targeted analysis of *p*-cresol and butyrate producing genes was not used, so it is unclear whether the absence of relative abundance values for uricase, tyrosine ammonia lyase (*thiH*), and hydroxyphenylacetate decarboxylase (*hpd*) genes is attributable to the untargeted analytical method or to actual undetectable levels of these genes. Notably, only 324 microbial species passed filtering, which must meet the threshold of 10+ reads in 3+ samples. Greater coverage in future missions will be needed to address the microbial species and community structure in more depth. In addition, the fecal proteome was not assessed in this study, so it was not possible to report whether the enzymes associated with these genes were also altered in space.

Ninth, the absence of detailed dietary data limits the ability to draw conclusions on the impact of dietary protein, tyrosine, and fiber intake on *p*-cresol dynamics, though TW’s energy intake at 66% of predicted requirements does raise concerns about inadequate intake. Based on historical ISS reporting, protein intake has generally been considered adequate on missions. Meanwhile, menus have not provided adequate fiber, nor have crew members selected adequate amounts of fiber (Smith, 2021). Future studies of *p*-cresol in space will have to more carefully quantify dietary intake.

Tenth, our study did not include access to clinical data that would have informed us about whether antibiotics were used Inflight and their potential impact on the results reported herein. Since numerous antibiotics have an adverse impact on gut microbial community structure and on *p*-cresol producers such as *Clostridioides difficile* (Miller et al., 2023), such data will be important for researchers in the future and for flight surgeons contemplating antibiotic use in space.

Finally, it bears noting that environmental exposure to toluene can also lead to *p*-cresol formation (Woiwode and Drysch, 1981). In our study, no toluene was detected in plasma, which suggests toluene is not a contributing factor to the *p*-cresol results reported herein. Also, toluene measures in ISS ambient air were not obtained. To our knowledge, “the only study of toluene levels in the ISS was a chemical analysis of the air filters following many years of use via a single timepoint” (JA Jones, personal communication, January 15, 2026), which would represent an accumulation time course of many years not reflective of a one-year exposure. The absence of ambient toluene data, however, leaves this potential confounder an open question for future investigation.

### 4.7 In closing

We report here the first effort to characterize *p*-cresol and its associated molecular dynamics in a long-duration human space mission (Garrett-Bakelman et al., 2019; Schmidt et al., 2020) and explore the concept of countermeasures for the purpose of new hypothesis generation and/or precision medicine applications. The degree to which the present finding of elevated *p*-cresol during 340 days in low Earth orbit is repeatable or accurately reflects the biological dynamics that would emerge from a repeated LEO exposure, prolonged lunar habitation, or a mission to Mars is currently unknown. Also unknown is whether there are any benefits to low concentrations of *p*-cresol in humans, though limited clinical and pre-clinical evidence has been reported (Brial et al., 2020).

Future exploration-class missions will benefit from targeted analysis of the *p*-cresol-associated molecular networks, with careful attention to the gut microbiome and to the phenotypic changes in astronauts. Given the substantial biological impact of *p*-cresol on systems relevant to human spaceflight, follow up examination of this phenomenon is strongly recommended.

Regarding precision medicine applications today, each of the measures reported here are presently quantifiable in astronauts and can be examined for their potential impact on astronaut training, mission planning, mission execution, and post-mission recovery. The associated countermeasures are readily available to practicing flight surgeons and astronauts today, though further research is clearly indicated. To these latter points, as the present paper was going to press, La Barbara et al. reported on gut derived metabolites from the plasma metabolome of 52 astronauts across mission types from 2-9 months duration. Indoxyl sulfate, 4-ethylphenol sulfate, and *p*-cresol sulfate, were elevated inflight compared with pre-flight (with *p*-cresol reaching statistical significance). Notably, there was substantial variability in *p*-cresol levels between astronauts. These follow up findings to our original report on *p*-cresol in space strengthens the case that *p*-cresol elevation is of importance in space and that the considerable variability between astronauts further strengthens the case for a precision medicine approach (La Barbera et al., 2026).

## Data Availability

The datasets presented in this study can be found in online repositories. The names of the repository/repositories and accession number(s) can be found below: https://soma.weill.cornell.edu/apps/SOMA_Browser.

## Supplementary material

Supplementary Figure 1 White blood cell RNA transcript profile for primary sulfotransferase genes (SULT1A1, SULT2A1, SULT1A3, SULT1A4).

Supplementary Figure 2 White blood cell RNA transcript profile for CYP4502E1.

Supplementary Figure 3 White blood cell RNA transcript profile for glutathione-S-transferase P1.
