# Correction: Systemic elevation of gut derived *p*-cresol during one year in space: implications for brain, heart, bone, liver and microbiome in astronauts

**DOI:** 10.3389/fphys.2026.2034151

**Published:** 2026-10-27

**Authors:** Michael A. Schmidt, Caleb M. Schmidt, Julian C. Schmidt, Cem Meydan, Evan E. Afshin, Eliah G. Overbey, JangKeun Kim, Ruth Subhash Singh, Afshin Beheshti, Christopher E. Mason

**Affiliations:** 1 Sovaris Aerospace, Boulder, CO, United States; 2 Department of Medicine, Aerospace Medicine, University of Central Florida College of Medicine, Orlando, FL, United States; 3 Center for Aerospace and Extreme Environments Medicine, UCF College of Medicine, Orlando, FL, United States; 4 Department of Systems Engineering, Colorado State University, Fort Collins, CO, United States; 5 Division of Hematology and Medical Oncology, Sanford IWeill Department of Medicine, Weill Cornell Medicine, New York, NY, United States; 6 Department of Physiology and Biophysics, Weill Cornell Medicine, New York, NY, United States; 7 The HRH Prince Alwaleed Bin Talal Bin Abdulaziz Alsaud Institute for Computational Biomedicine, Weill Cornell Medicine, New York, NY, United States; 8 Center for STEM, University of Austin (UATX), Austin, TX, United States; 9 Department of Biomedical Engineering, Rensselaer Polytechnic Institute, Troy, NY, United States; 10 Stanley Center for Psychiatric Research, Broad Institute of MIT and Harvard, Cambridge, MA, United States; 11 Center for Space Biomedicine, Trivedi Institute for Space and Global Biomedicine, University of Pittsburgh School of Medicine, Pittsburgh, PA, United States; 12 McGowan Institute for Regenerative Medicine, Department of Surgery, University of Pittsburgh, Pittsburgh, PA, United States; 13 Computational and Systems Biology, University of Pittsburgh School of Medicine, Pittsburgh, PA, United States; 14 Trivedi Institute for Space and Global Biomedicine, University of Pittsburgh School of Medicine, Pittsburgh, PA, United States; 15 The WorldQuant Initiative for Quantitative Prediction, Weill Cornell Medicine, New York, NY, United States; 16 The Feil Family Brain and Mind Research Institute, Weill Cornell Medicine, New York, NY, United States

**Keywords:** microbiome, multiomics, NASA twins study, *p*-cresol, precision medicine, spaceflight, metabolome, prebiotics

The **Ethics statement** was erroneously given as “The studies involving humans were approved by Weill Cornell Medicine Institution Review Board (WCM-IRB), NASA Institutional Review Board. The correct **Ethics statement** is “The studies involving humans were approved by the NASA Institutional Review Board for the NASA Twins Study.”

The **Data Availability statement** was erroneously given as “https://soma.weill.cornell.edu/apps/SOMA_Browser/2020.1.”. The correct **Data Availability statement** is “https://soma.weill.cornell.edu/apps/SOMA_Browser”.

The original version of this article has been updated.

